# Comparing Efficacy and Safety of Various Monoclonal Antibodies in Myasthenia Gravis: A Systematic Review and Network Meta‐Analysis of Randomized Controlled Trials

**DOI:** 10.1002/brb3.71557

**Published:** 2026-06-16

**Authors:** Muhammad Hassan Waseem, Zain ul Abideen, Minahil Iqbal, Saniya Ishtiaq, Pawan Kumar Thada, Adam A. Dmytriw

**Affiliations:** ^1^ Allama Iqbal Medical College Lahore Pakistan; ^2^ King Edward Medical University Lahore Pakistan; ^3^ Rawalpindi Medical University Rawalpindi Pakistan; ^4^ Sotang Primary Hospital Solukhumbu Nepal; ^5^ Neuroendovascular Program, Massachusetts General Hospital & Brigham and Women's Hospital Harvard Medical School Boston Massachusetts USA; ^6^ Neurointerventional & Neuroanalytics Collaboration (NAN‐C), School of Medicine Toronto Metropolitan University Toronto Canada; ^7^ Nuffield Department of Surgical Sciences, Medical Sciences Division University of Oxford Oxford UK

**Keywords:** complement inhibitor, FcRn inhibitor, monoclonal antibodies, myasthenia gravis, network meta‐analysis

## Abstract

**Background:**

Myasthenia gravis (MG) is an autoimmune disorder that causes muscle weakness due to disrupted neuromuscular transmission. This network meta‐analysis compared the safety and effectiveness of monoclonal antibodies used to treat MG.

**Methods:**

PubMed, Cochrane Central, and ScienceDirect were searched through June 2025. A frequentist network meta‐analysis was conducted using the “meta” and “netmeta” packages in RStudio version 4.3.3. Treatment rankings were determined by *p*‐scores.

**Results:**

Eighteen randomized controlled trials (RCTs) were included in this network meta‐analysis. Rozanolixzumab (ROZ) 10 mg/kg significantly decreased the MG Activities of Daily Living (MG‐ADL) score compared to placebo (MD = −2.33; 95%CI:[‐3.60, −1.06]; *p* = 0.0003) and was ranked best (*p*‐score = 0.77) regarding this outcome. Batoclimab (BAT) 680 mg significantly reduced the Quantitative MG (QMG) score compared to placebo (MD = ‐5.17; 95%CI:[‐6.44, ‐3.89]; p <0.0001) and was rated the top performer (*p*‐score = 0.96) for this endpoint. Regarding the MG Composite (MGC) score, BAT 340 mg was ranked best (*p*‐score = 0.82), although the decrease in MGC score was insignificant (MD −5.30; 95%CI:[−11.01, 0.41]; *p* = 0.07). Eculizumab (ECU) was ranked best (*p*‐score = 0.93) regarding the 15‐item revised MG Quality of Life (MG‐QoL 15r) score. Belimumab (BEL) 10 mg/kg was ranked best regarding the adverse events (*p*‐score = 0.87).

**Conclusion:**

ROZ 10 mg/kg ranked best for MG‐ADL. BAT 680 mg significantly reduced QMG and ranked best. BAT 340 mg had the highest probability of reducing the MGC score. ECU significantly decreased MG‐QoL 15r and ranked best. BEL 10 mg/kg had the highest likelihood of reducing adverse events.

## Graphical abstract

ROZ 10 mg/kg ranked best for reducing MG‐ADL. BAT 680 mg significantly reduced QMG and ranked best. BAT 340 mg had the highest probability of reducing the MGC score. ECU significantly decreased MG‐QoL 15r and ranked best. BEL 10 mg/kg had the highest likelihood of reducing adverse events.

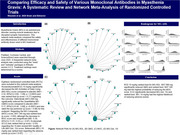

## 1 Introduction

Myasthenia gravis (MG) is a chronic, autoimmune neuromuscular disorder characterized by fluctuating skeletal muscle weakness due to impaired synaptic transmission at the neuromuscular junction (NMJ). This dysfunction is primarily caused by IgG autoantibodies targeting the nicotinic acetylcholine receptor (AChR), muscle‐specific kinase (MuSK), or low‐density lipoprotein receptor‐related protein 4 (LRP4) (Gilhus et al. 2019; Gilhus and Verschuuren 2015; Pevzner et al. 2012). The clinical presentation ranges from pure ocular symptoms to generalized weakness affecting bulbar, limb, and respiratory muscles, with significant variability in severity and progression. Globally, MG affects approximately 150–250 individuals per million (Gilhus et al. 2019). There is a decreasing incidence of MG likely due to heightened awareness and improved diagnostic capabilities (Carr et al. 2010; Witthayaweerasak et al. 2021).

Conventional MG treatments include cholinesterase inhibitors (e.g., pyridostigmine), corticosteroids, and non‐steroidal immunosuppressants such as azathioprine, mycophenolate mofetil, and cyclosporine (Narayanaswami et al. 2021; Dalakas 2019; Guptill et al. 2016). Although many patients experience symptomatic improvement, up to 20% remain refractory or experience intolerable side effects, such as osteoporosis, hypertension, diabetes, and increased infection or malignancy risk (Drachman 1994; Mantegazza and Antozzi 2018). In addition, these immunosuppressive therapies are non‐specific and often require months to take effect, limiting their utility in rapidly progressive disease (Howard et al. 2017).

Advances in the understanding of MG immunopathogenesis have led to the development of monoclonal antibody‐based therapies targeting specific immune pathways. These include complement inhibitors such as eculizumab (ECU), ravulizumab (RAV), and zilucoplan (ZIL) that block complement component C5 to prevent membrane damage at the NMJ (Vu et al. 2025; Howard et al. 2021, 2023), as well as neonatal Fc receptor (FcRn) antagonists like efgartigimod (EFG) and rozanolixizumab (ROZ) that reduce circulating IgG autoantibody levels by inhibiting their recycling (Habib et al. 2024; Shi et al. 2024). While these therapies have demonstrated clinical efficacy in recent trials, treatment selection remains empirical and lacks comparative guidance.

Furthermore, disease subtypes appear to influence biologic response. MuSK‐positive patients often respond better to rituximab (RIT) (an anti‐CD20 monoclonal antibody), while AChR‐positive patients show more significant benefit from complement or FcRn‐targeting agents (Hehir and Silvestri 2018). Despite promising results, the growing number of monoclonal agents, each with different mechanisms and dosing regimens, has complicated clinical decision‐making.

No study has systematically compared the dosing regimens of these monoclonal antibodies across studies to date. The present network meta‐analysis addresses this gap by being the first attempt to evaluate and compare the dosage strategies of monoclonal antibodies used in MG. In addition, the recent publication of high‐quality Phase 3 trials has enriched the available evidence base, providing an opportunity for updated synthesis. Finally, two novel biologics, including satralizumab (SAT) (IL‐6 receptor inhibitor) and inebilizumab (INE) (anti‐CD19 agent), have recently entered the therapeutic landscape for MG, further emphasizing the need for a comprehensive, comparative evaluation. The current study focuses on synthesizing data from existing randomized controlled trials (RCTs) to compare efficacy and safety and to rank dosing regimens for monoclonal antibodies, thereby offering useful insights to clinicians navigating this rapidly evolving landscape of MG treatment.

## 2 Methods

This systematic review and network meta‐analysis followed the Preferred Reporting Items for Systematic Reviews and Meta‐Analyses (PRISMA) statement (Page et al. 2021) and is in accordance with the Cochrane Handbook for Systematic Reviews of Interventions (Higgins et al. 2019). The protocol for this review was registered with PROSPERO under the ID CRD420251129249.

### 2.1 Literature Search

A detailed search was conducted across electronic databases, including PubMed, Cochrane Library, and ScienceDirect, from inception to June 2025. The bibliographies of the included studies were searched for any relevant articles. The MeSH terms and keywords used included: “Myasthenia Gravis,” “Generalized Myasthenia Gravis,” “Muscle‐Specific Receptor Tyrosine Kinase Myasthenia Gravis,” “MuSK Myasthenia Gravis,” “Antibodies, Monoclonal,” “Fc receptor, neonatal,” “Complement Inactivating Agents,” and “Antigens, CD.” The details of search strings used in different electronic databases are provided in Table S1.

### 2.2 Study Selection and Eligibility Criteria

**FIGURE 1:**
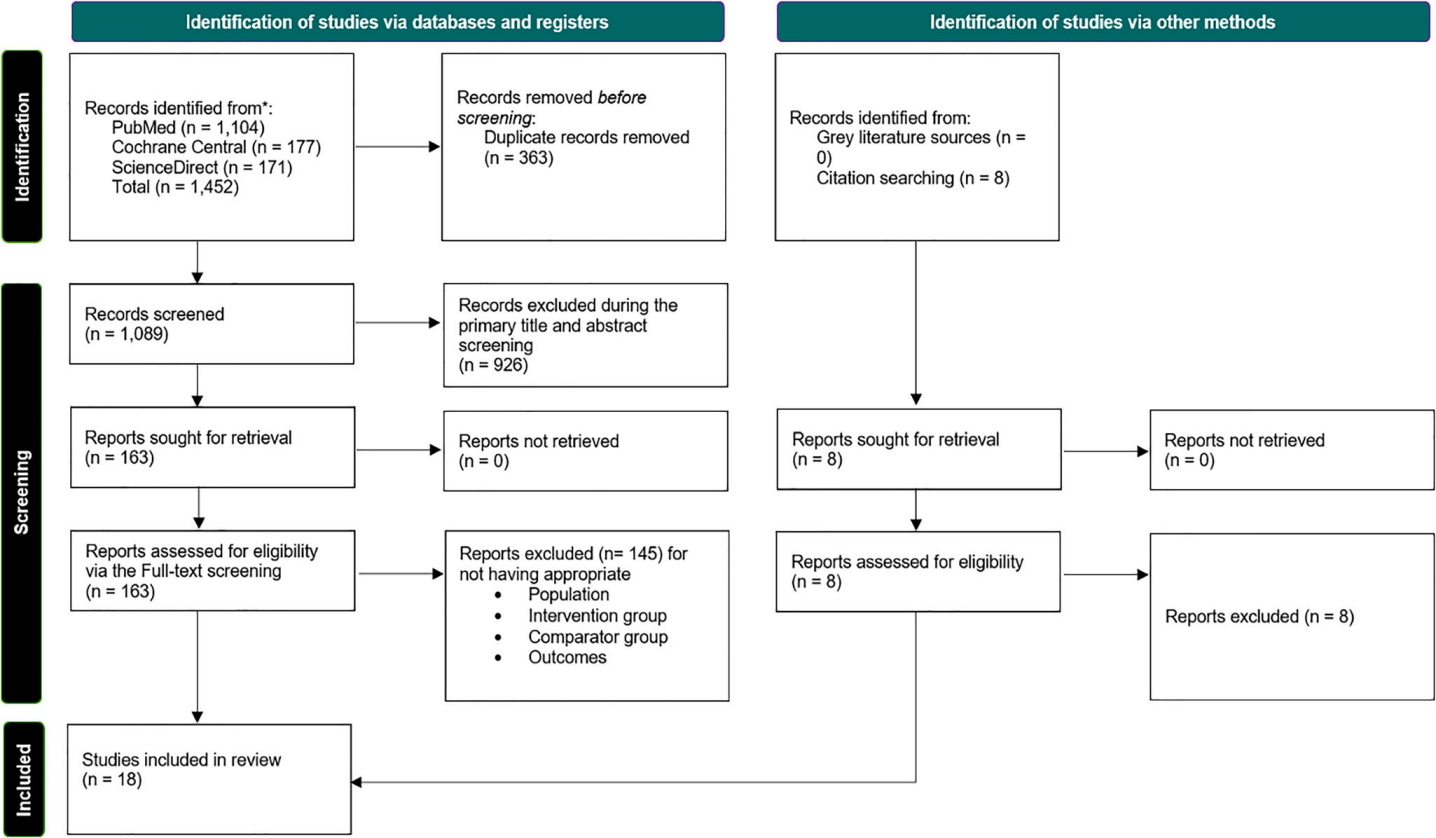
PRISMA flowchart of the study selection process.

The articles obtained from searching the targeted databases underwent initial screening of titles and abstracts after duplicates were removed. Those articles that passed this primary screening then proceeded to a secondary full‐text review. Two independent researchers (M.I. and Z.U.A.) conducted the screening, and any disagreements were resolved by a third author (M.H.W.). The PRISMA flowchart of the study selection process is shown in Figure 1.

The inclusion criteria encompassed adult patients aged 18 years or older with a confirmed diagnosis of generalized MG, classified as clinical Class II–V according to the Myasthenia Gravis Foundation of America (MGFA). Patients in the intervention group received various doses of monoclonal antibodies, while those in the control group received a placebo. Studies were included if they reported at least one of the following outcomes: MG Activities of Daily Living (MG‐ADL), Quantitative MG (QMG), MG Composite (MGC), the 15‐item revised MG Quality of Life (MG‐QoL 15r) scores, or adverse events. The study design included only RCTs.

Patients under 18, studies that did not report the relevant intervention and comparison groups, studies lacking the desired outcomes, and study designs such as case reports, case series, observational studies, and reviews were excluded.

### 2.3 Data Extraction and Endpoints Definitions

Two independent authors (Z.U.A. and S.I.) extracted the data on a predefined Microsoft Excel sheet, with any discrepancies resolved by a third author (M.H.W.). The extracted baseline characteristics included the author's name, year, trial phase, sample size, gender distribution, mean age, monoclonal antibody classes used, thymectomy incidence, and presence of AChR+ MG patients.

The extracted endpoints were MG‐ADL, QMG, MGC, MG‐QoL 15r scores, and adverse events. The MG‐ADL is an eight‐item scale with a score range from 0 to 24 points, used to evaluate how MG symptoms impact daily activities (Wolfe et al. 1999). The QMG is a 13‐item scale with scores ranging from 0 to 39, based on quantitative testing of the sentinel muscle groups (Barohn 2025). The MGC is a 10‐item scale with scores from 0 to 50 that assesses the clinical status of MG patients by evaluating symptoms of the disease (Burns et al. 2010). The MG‐QoL 15 is a 15‐item questionnaire with scores from 0 to 60, designed to assess various aspects of daily life quality in MG patients (Burns et al. 2016). The revised scale (MG‐QoL 15r) is used in all the included studies except the study by Howard et al. (2017).

### 2.4 Bias Assessment

The risk of bias of the included RCTs was assessed using the Cochrane Risk of Bias (RoB 2.0) tool (Sterne et al. 2019). The RoB 2.0 tool evaluates quality across five domains: (1) Bias from the randomization process, (2) bias from deviations in the intended intervention, (3) bias caused by missing outcome data, (4) bias in how outcomes are measured, and (5) bias in selecting which results to report. The risk of bias for each included study was classified as high, low, or uncertain.

### 2.5 Statistical Analysis

A frequentist network meta‐analysis was conducted using the “meta” and “netmeta” packages in RStudio version 4.3.3, employing a random‐effects model. The risk ratios (RRs) and mean differences (MDs), together with 95% confidence intervals (CIs), were combined to evaluate the network effects for dichotomous and continuous outcomes, respectively. The placebo group served as the reference. The studies were included following the transitivity criteria. Statistical significance was determined at *p* < 0.05. Heterogeneity was evaluated using *τ* ^2^ and *I* ^2^ values. Node‐splitting analysis to detect inconsistencies between direct and indirect estimates was conducted only when closed loops in the network were identified; otherwise, it was not performed. When data were not provided as standard deviation (SD) but rather as ranges, interquartile ranges (IQRs), or standard error of the mean (SEM), they were converted to SD using methods established by Wan et al. (2014). Network plots were created using NMA Studio to visualize the comparison, where each node represents an intervention, and the thickness of the lines between nodes indicates the number of RCTs comparing the two interventions. Each intervention's efficacy and safety were ranked using *p*‐scores, where a *p*‐score of 1 indicates the highest safety or effectiveness, and a *p*‐score of 0 indicates the lowest. Rankograms were used to depict intervention rankings based on *p*‐scores, and league tables were created to compare the effects of various interventions. Publication bias was assessed visually through funnel plots and statistically via Egger's regression test.

## 3 Results

### 3.1 Search Results

When searching various databases, including PubMed, Cochrane Central, and ScienceDirect, from inception to June 2025, a total of 1452 potential articles were identified. After removing 363 duplicates, the remaining 1089 articles underwent initial screening of titles and abstracts, resulting in 163 articles. These were then subjected to a detailed full‐text review, which ultimately led to the inclusion of 18 articles (Howard et al. 2017, 2019, 2020, 2021, 2023; Antozzi et al. 2024, 2025; Bril et al. 2021, 2023; Habib et al. 2025; Hewett et al. 2018; GomezMancilla et al. 2024; Nowak et al. 2022, 2025; Piehl et al. 2022; Vu et al. 2022; Yan et al. 2022, 2024) in this systematic review and network meta‐analysis.

### 3.2 Study Characteristics

Eighteen RCTs (Howard et al. 2017, 2019, 2020, 2021, 2023; Antozzi et al. 2024, 2025; Bril et al. 2021, 2023; Habib et al. 2025; Hewett et al. 2018; GomezMancilla et al. 2024; Nowak et al. 2022, 2025; Piehl et al. 2022, Vu et al. 2022; Yan et al. 2022, 2024) published between 2017 and 2025 were included in this network meta‐analysis. The mean age ranged from 36 to 67 years. The monoclonal antibodies used were ROZ at 7 and 10 mg/kg, batoclimab (BAT) at 340 and 680 mg, EFG at 10 mg/kg, ECU, ZIL at 0.1 mg/kg and 0.3 mg/kg, RAV, belimumab (BEL) at 10 mg/kg, RIT, iscalimab (ISC) at 10 mg/kg, INE, SAT, nipocalimab (NIP) at 5 mg/kg Q4W (once every 4 weeks), 30 mg/kg Q4W (once every 4 weeks), 60 mg/kg as a single dose, 60 mg/kg Q2W (once every 2 weeks), and 30/15 mg/kg Q2W (a loading dose of 30 mg/kg followed by 15 mg/kg every 2 weeks for maintenance). The number of patients who underwent thymectomy varied from 2 to 59, while the incidence of AChR+ MG patients ranged from 3 to 95. The classes of monoclonal antibodies include FcRn inhibitors, complement inhibitors, and those targeting CD20, CD19, CD40, and IL‐6 receptor. Details of the baseline characteristics of the included studies are provided in Table 1.

**TABLE 1:** Baseline characteristics of the included studies.

Study ID	Phase	Sample size	Gender (M/F)	Mean age (years)	INT	Time since onset (years)	Thymectomy	INT periods	AChR+	Ab type
Yan 2024	3	BAT: 67 PLA: 64	BAT: 27/40 PLA:16/48	BAT: 43.8 ± 13.9 PLA: 43.7 ± 13.5	BAT	—	BAT: 23 PLA: 14	6 weeks	BAT: 65 PLA: 59	FcRn inhibitor
Yan 2022	2	BAT 680 mg: 11 BAT 340 mg: 10 PLA: 9	BAT 680 mg: 2/9 BAT 340 mg: 2/8 PLA: 2/7	BAT 680 mg: 40.6 ± 16.8 BAT 340mg: 36.4 ± 9.8 PLA: 40.2 ± 9.3	BAT	BAT 680 mg: 6.4 ± 5.7 BAT 340 mg: 9.8 ± 10.8 PLA: 6.0 ± 6.8	BAT 680 mg: 3 BAT 340 mg: 3 PLA: 2	43 days	BAT 680 mg: 11 BAT 340 mg: 9 PLA: 8	FcRn inhibitor
Piehl 2022	3	RIT: 25 PLA: 22	RIT: 18/7 PLAa: 15/7	RIT: 67.4 ± 13.4 PLA: 58 ± 18.6	RIT	—	—	16 weeks	RIT: 23 PLA: 22	Targets CD20
Vu 2022	3	RAV: 86 PLA: 89	RAV: 42/44 PLA: 44/45	RAV: 58.0 ± 13.8 PLA: 53.3 ± 16.1	RAV	RAV: 9.8 ± 9.7 PLA: 10.0 ± 8.9	—	26 weeks	RAV: 86 PLA: 89	Complement inhibitor
Nowak 2025	3	INE: 119 PLA: 117	INE: 54/65 PLA: 38/79	INE: 47.1 ± 15.7 PLA: 47.9 ± 15.0	INE	INE: 58.0 ± 13.8 PLA: 53.3 ± 16.1	INE: 39 PLA: 32	26 weeks	INE: 95 PLA: 93	Targets CD19
Nowak 2022	2	RIT: 25 PLA: 27	RIT: 14/11 PLA: 15/12	RIT: 53.2 ± 17.5 PLA: 56.8 ± 17	RIT	—	RIT: 8 PLA: 14	52 weeks	RIT: 25 PLA: 27	Targets CD20
Mancilla 2024	2	ISC: 22 PLA: 22	ISC: 10/12 PLA: 6/16	ISC: 44.7 ± 13.5 PLA: 43.3 ± 13.9	ISC	ISC: 8.2 ± 6.96 PLA: 8.4 ± 8.47	—	25 weeks	ISC: 22 PLA: 22	Targets CD40
Howard 2023	3	ZIL: 86 PLA: 88	ZIL: 34/52 PLA: 41/47	ZIL: 52.6 ± 14.6 PLA: 53.3 ± 15.7	ZIL	ZIL: 9.3 ± 9.5 PLA: 9.0 ± 10.4	ZIL: 45 PLA: 37	12 weeks	ZIL: 86 PLA: 88	Complement inhibitor
Howard 2021	3	EFG: 84 PLA: 83	EFG: 21/63 PLA: 28/55	EFG: 45.9 ± 14.4 PLA: 48.2 ± 15	EFG	EFG: 10.1 ± 9 PLA: 8.8 ± 7.6	EFG: 59 PLA: 36	8 weeks	EFG: 65 PLA: 64	FcRn inhibitor
Howard 2020	2	ZIL 0.1 mg/kg: 15 ZIL 0.3 mg/kg: 14 PLA:15	ZIL 0.1 mg/kg:7/8 ZIL 0.3 mg/kg:10/4 PLA: 4/11	ZIL 0.1 mg/kg: 45.5 ± 15.7 ZIL 0.3 mg/kg: 54.6 ± 15.5 PLA: 48.4 ± 15.7	ZIL	ZIL 0.1 mg/kg: 6.5 ± 5.63 ZIL 0.3 mg/kg: 5.3 ± 6.38 PLA: 6.3 ± 5.2	ZIL 0.1 mg/kg: 8 ZIL 0.3 mg/kg: 7 PLA:5	12 weeks	ZIL 0.1 mg/kg: 15 ZIL 0.3 mg/kg: 14 PLA: 15	Complement inhibitor
Howard 2019	2	EFG: 12 PLA: 12	EFG :5/7 PLA: 4/8	EFG: 55.3 ± 13.6 PLA: 43.5 ± 19.3	EFG	EFG: 8.2 ± 9 PLA: 13.3 ± 11.2	—	78 days	—	FcRn inhibitor
Howard 2017	3	ECU: 62 PLA: 63	ECU: 21/41 PLA: 22/41	ECU: 47.5 ± 15.7 PLA: 46.9 ± 18	ECU	ECU: 9.9 ± 8.1 PLA: 9.2 ± 8.4	ECU: 37 PLA: 31	26 weeks	—	Complement inhibitor
Hewett 2018	2	BEL: 18 PLA: 21	BEL: 8/10 PLA: 7/14	BEL: 52.7 ± 17.32 PLA: 59 ± 13.88	BEL	BEL: 6.95 ± 9.03 PLA: 8.30 ± 8.06	BEL: 6 PLA: 7	24 weeks	BEL: 18 PLA: 20	B‐lymphocyte stimulator specific inhibitor
Habib 2025	3	SAT: 96 PLA: 92	SAT: 33/63 PLA: 36/56	SAT: 47.0 ± 14.4 PLA: 45.9 ± 16.3	SAT	SAT: 8.8 ± 7.7 PLA: 8.5 ± 9.3	SAT: 30 PLA: 33	24 weeks	SAT: 86 PLA: 82	Targets IL‐6 receptor
Bril 2023	3	ROZ 7 mg/kg: 66 ROZ 10 mg/kg: 67 PLA: 67	ROZ 7 mg/kg: 27/39 ROZ 10 mg/kg: 32/35 PLA: 20/47	ROZ 7 mg/kg: 53.2 ± 14.7 ROZ 10 mg/kg: 51.9 ± 16.5 PLA: 50.4 ± 17.7	ROZ	—	ROZ 7 mg/kg: 32 ROZ 10 mg/kg: 20 PLA: 31	43 days	ROZ 7 mg/kg: 60 ROZ 10 mg/kg: 60 PLA: 59	FcRn inhibitor
Bril 2021	2	ROZ: 21 PLA: 22	ROZ: 8/13 PLA: 8/14	ROZ: 50.5 ± 14.7 PLA: 53 ± 15.7	ROZ	—	ROZ: 11 PLA: 10	29 days	ROZ: 19 PLA: 21	FcRn inhibitor
Antozzi 2024	2	NIP: 54 PLA: 14	NIP: 25/29 PLA: 6/8	NIP: 57.5 ± 14.8 PLA: 60.5 ± 14.5	NIP	NIP: 7.3 ± 7.31 PLA: 13.2 ± 9.81	—	57 days	NIP: 51 PLA: 3	FcRn inhibitor
Antozzi 2025	3	NIP: 77 PLA: 76	NIP: 27/50 PLA: 34/42	NIP: 52.5 ± 15.66 PLA: 52.3 ± 16.37	NIP	NIP: 6.9 ± 7.44 PLA: 8.90 ± 8.13	—	24 weeks	NIP: 49 PLA: 54	FcRn inhibitor

Abbreviations: Ab = Antibody; AChR+ = Nicotinic acetylcholinic receptor positive; BAT = Batoclimab; BEL = Belimumab; ECU = Eculizumab; EFG = Efgartigimod; FcRn = Neonatal Fc receptor; INE = Inebilizumab; INT = Intervention; ISC = Iscalimab; NIP = Nipocalimab; PLA = Placebo. RAV = Ravulizumab; RIT = Rituximab; ROZ = Rozanolixzumab; SAT = Satralizumab; ZIL = Zilucoplan.

### 3.3 Risk of Bias

**FIGURE 2:**
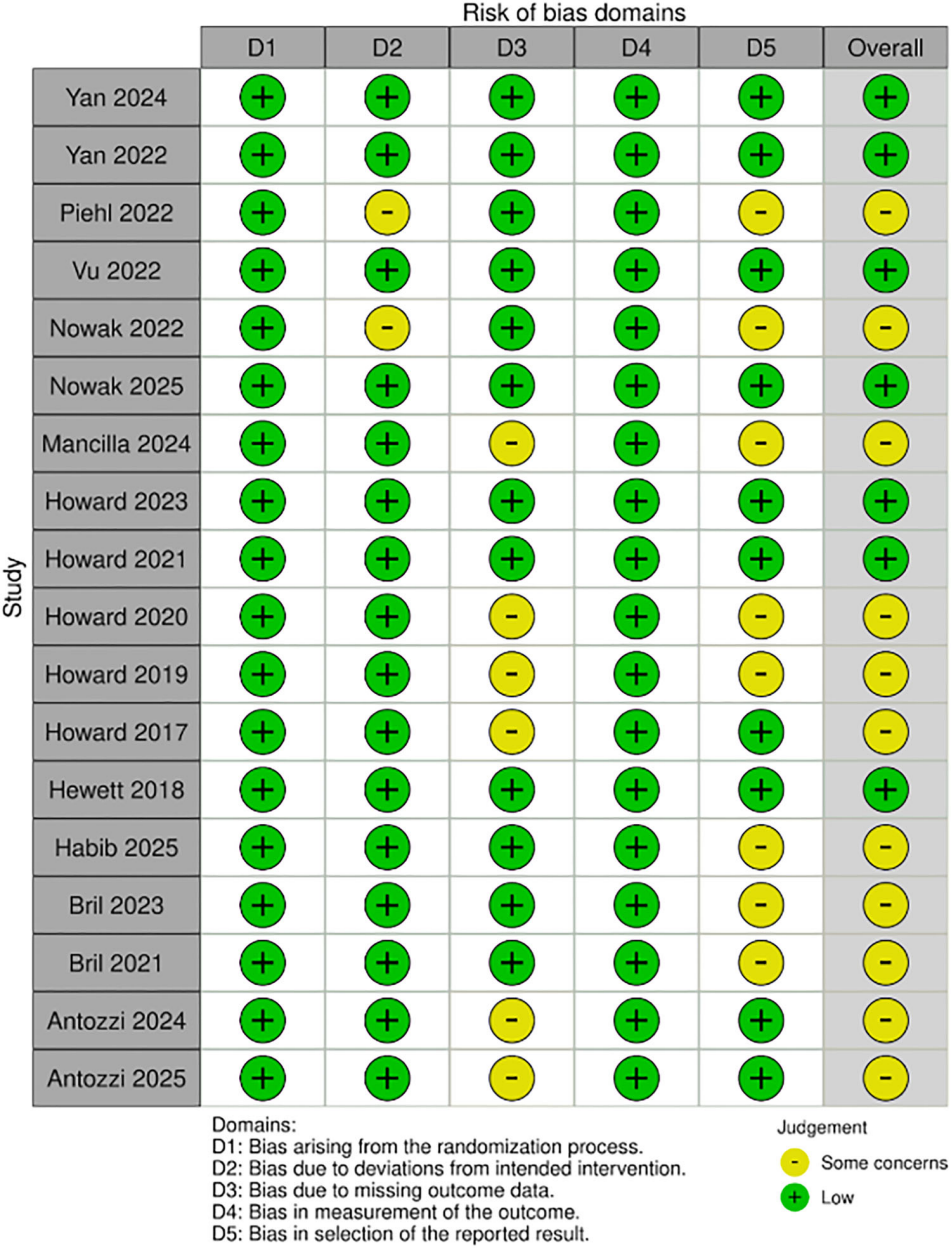
The Cochrane risk of bias (RoB 2.0) tool traffic light plot.

The risk of bias of the included RCTs was assessed using the Cochrane RoB 2.0 tool (Sterne et al. 2019). Seven of the included RCTs have a low risk of bias (Howard et al. 2021, 2023; Hewett et al. 2018; Nowak et al. 2025; Vu et al. 2022; Yan et al. 2022, 2024), whereas the rest have some concerns. The traffic light plot of the risk of bias is depicted in **Figure** **2**.

### 3.4 Network Meta‐Analysis

#### 3.4.1 MG‐ADL

**FIGURE 3:**
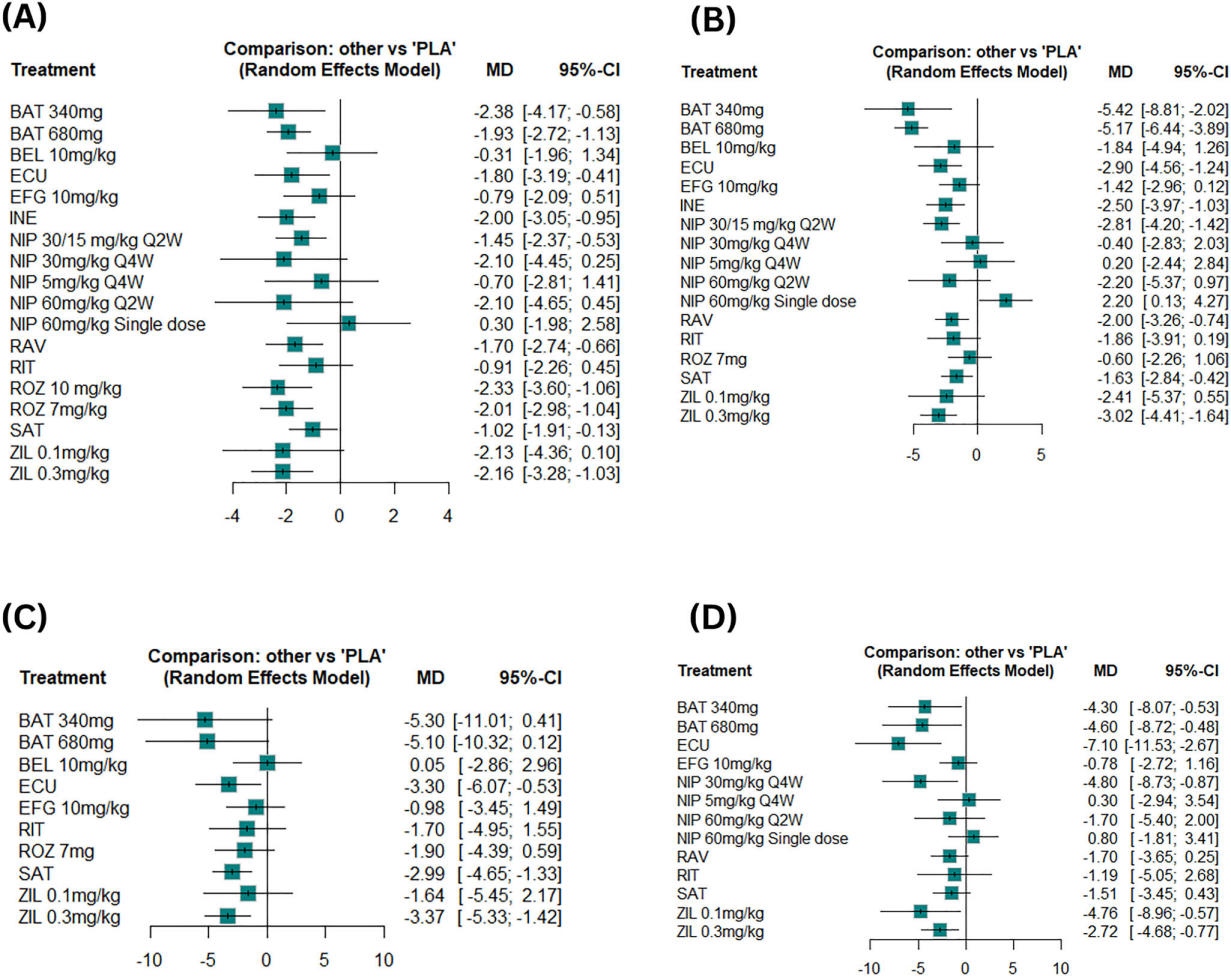
Forest plots for (A) myasthenia gravis activities of daily living (MG‐ADL) score, (B) quantitative myasthenia gravis (QMG) score, (C) myasthenia gravis composite (MGC) score, (D) 15‐item revised myasthenia gravis quality of Life (MG‐QoL 15r) score; (BAT: Batoclimab; BEL: Belimumab; CI: Confidence interval; ECU: Eculizumab; EFG: Efgartigimod; INE: Inebilizumab; MD: Mean difference; NIP: Nipocalimab; PLA: Placebo; RAV: Ravulizumab; RIT: Rituximab; ROZ: Rozanolixzumab; SAT: Satralizumab; ZIL: Zilucoplan).

**FIGURE 4:**
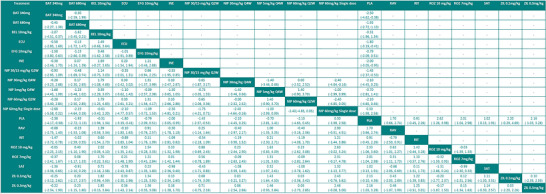
League table for myasthenia gravis activities of daily living (MG‐ADL) score (BAT: Batoclimab; BEL: Belimumab; ECU: Eculizumab; EFG: Efgartigimod; INE: Inebilizumab; NIP: Nipocalimab; PLA: Placebo; RAV: Ravulizumab; RIT: Rituximab; ROZ: Rozanolixzumab; SAT: Satralizumab; ZIL: Zilucoplan).

**FIGURE 5:**
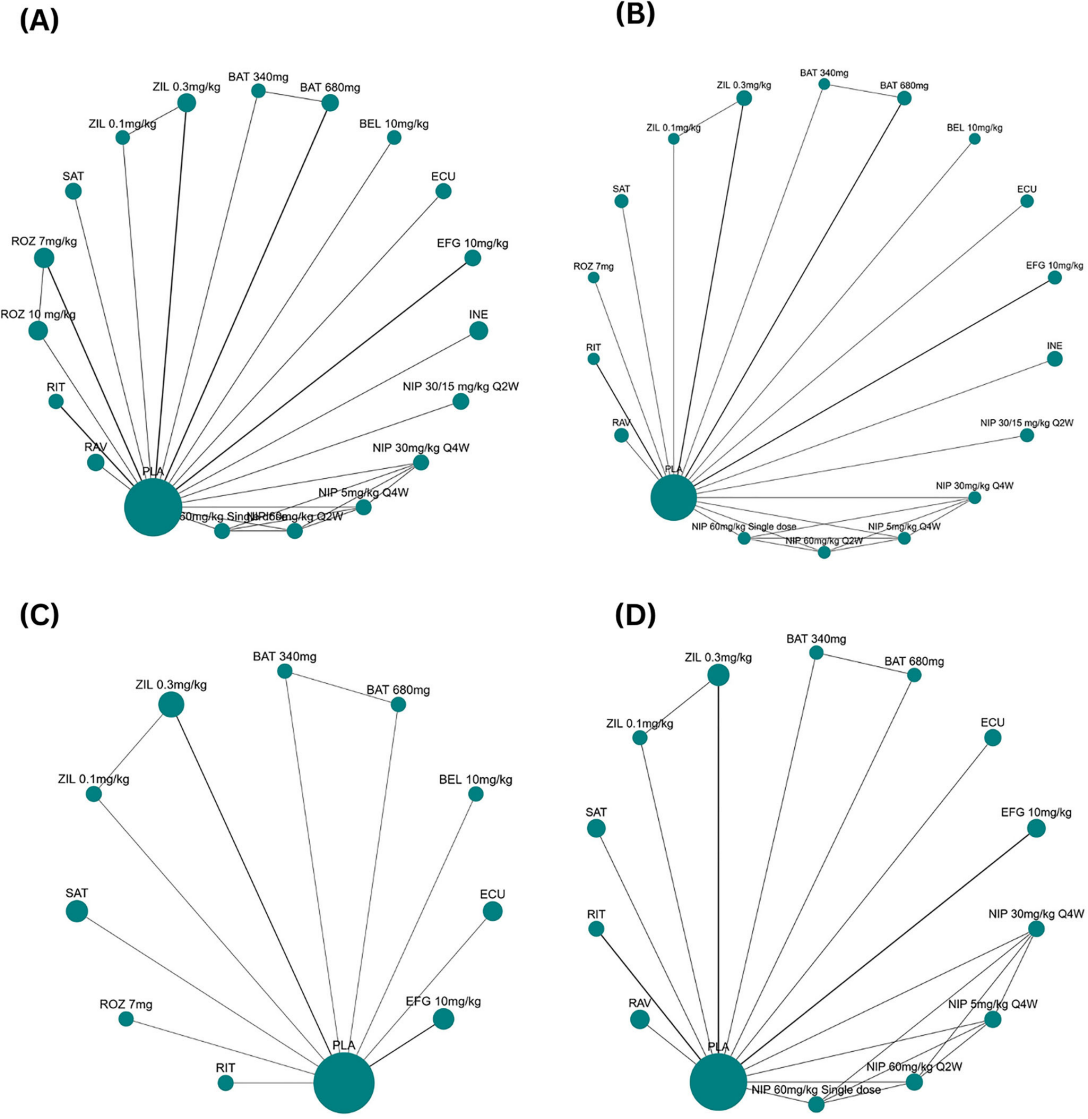
Network plots for (A) myasthenia gravis activities of daily living (MG‐ADL) score, (B) quantitative myasthenia gravis (QMG) score, (C) myasthenia gravis composite (MGC) score, (D) 15‐item revised myasthenia gravis quality of life (MG‐QoL 15r) score (BAT: batoclimab; BEL: belimumab; ECU: eculizumab; EFG: efgartigimod; INE: inebilizumab; NIP: nipocalimab; PLA: placebo; RAV: ravulizumab; RIT: rituximab; ROZ: rozanolixzumab; SAT: satralizumab; ZIL: zilucoplan).

**FIGURE 6:**
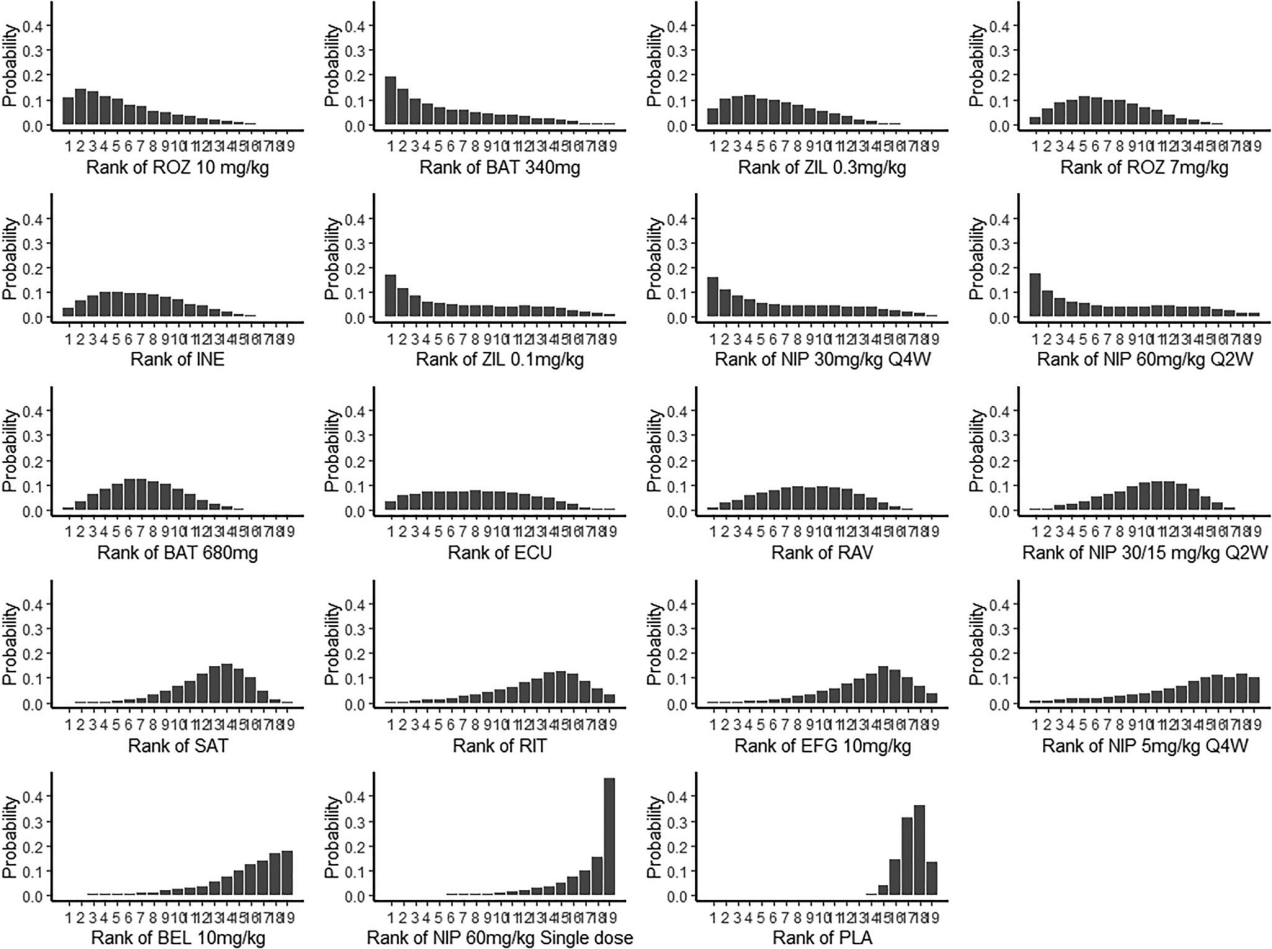
Rankogram based on *p*‐score for myasthenia gravis activities of daily living (MG‐ADL) score (BAT: Batoclimab; BEL: Belimumab; ECU: Eculizumab; EFG: Efgartigimod; INE: Inebilizumab; NIP: Nipocalimab; PLA: Placebo; RAV: Ravulizumab; RIT: Rituximab; ROZ: Rozanolixzumab; SAT: Satralizumab; ZIL: Zilucoplan).

The network meta‐analysis incorporated seventeen studies, involving 19 treatments and 32 pairwise comparisons for this outcome. Compared to placebo, BAT 340 mg (MD = −2.38; 95%CI:[−4.17, −0.58]; *p* = 0.009), BAT 680 mg (MD = −1.93; 95%CI:[−2.72, −1.13]; *p* <0.0001), ECU (MD = −1.80; 95%CI:[−3.19, −0.41]; *p* = 0.01), INE (MD = −2.00; 95%CI:[−3.05, −0.95]; *p* = 0.0002), NIP 30/15 mg/kg Q2W (MD = −1.45; 95%CI:[−2.37, −0.53]; *p* = 0.002), RAV (MD = −1.70; 95%CI:[−2.74, −0.66]; *p* = 0.001), ROZ 10 mg/kg (MD = −2.33; 95%CI:[−3.60, −1.06]; *p* = 0.0003), ROZ 7 mg/kg (MD = −2.01; 95%CI:[−2.98, −1.04]; *p* <0.0001), SAT (MD = −1.02; 95%CI:[−1.91, −0.13]; *p* = 0.02), and ZIL 0.3 mg/kg (MD = −2.16; 95%CI:[−3.28, −1.03]; *p* = 0.0002) significantly reduced the MG‐ADL score. BEL 10 mg/kg (*p* = 0.71), EFG 10 mg/kg (*p* = 0.23), NIP 30 mg/kg Q4W (*p* = 0.08), NIP 5 mg/kg Q4W (*p* = 0.51), NIP 60 mg/kg Q2W (*p* = 0.11), NIP 60 mg/kg single dose (*p* = 0.80), RIT (*p* = 0.19), and ZIL 0.1 mg/kg (*p* = 0.06) were comparable to placebo regarding this endpoint (Figures 3A and 4). The network diagram of this outcome is shown in Figure 5A. ROZ 10 mg/kg was ranked best (*p*‐score = 0.7680), whereas the NIP 60 mg/kg single dose was ranked worst (0.1079). The details regarding the treatment ranking are provided in Table 2 and Figure 6. No heterogeneity was detected among the included studies (*τ* ^2 ^< 0.0001, *I* ^2^ = 0%).

**TABLE 2:** *p*‐score ranking.

MG‐ADL		QMG		MGC		MG‐QoL 15r		Adverse events	
Intervention	*p*‐score	Intervention	*p*‐score	Intervention	*p*‐score	Intervention	*p*‐score	Intervention	*p*‐score
ROZ 10 mg/kg	0.7680	BAT 680 mg	0.9576	BAT 340 mg	0.8161	ECU	0.9257	BEL 10 mg/kg	0.8656
BAT 340 mg	0.7523	BAT 340 mg	0.9319	BAT 680 mg	0.8124	NIP 30 mg/kg Q4W	0.7903	EFG 10 mg/kg	0.8152
ZIL 0.3 mg/kg	0.7216	ZIL 0.3 mg/kg	0.7356	ZIL 0.3 mg/kg	0.7015	ZIL 0.1 mg/kg	0.7769	NIP 30 mg/kg Q4W	0.7651
ROZ 7 mg/kg	0.6754	ECU	0.7051	ECU	0.6723	BAT 680 mg	0.7627	ISC 10 mg/kg	0.7504
INE	0.6726	NIP 30/15 mg/kg Q2W	0.6949	SAT	0.6383	BAT 340 mg	0.7410	NIP 30/15 mg/kg Q2W	0.6374
ZIL 0.1 mg/kg	0.6701	INE	0.6309	ROZ 7 mg/kg	0.4483	ZIL 0.3 mg/kg	0.5908	BAT 680 mg	0.6151
NIP 30 mg/kg Q4W	0.6681	ZIL 0.1 mg/kg	0.5918	RIT	0.4188	NIP 60 mg/kg Q2W	0.4455	RIT	0.5186
NIP 60 mg/kg Q2W	0.6606	NIP 60 mg/kg Q2W	0.5601	ZIL 0.1 mg/kg	0.4098	RAV	0.4445	RAV	0.4892
BAT 680 mg	0.6519	RAV	0.5236	EFG 10 mg/kg	0.2981	SAT	0.4153	BAT 340 mg	0.4487
ECU	0.5991	RIT	0.4966	BEL 10 mg/kg	0.1618	RIT	0.3705	NIP 5 mg/kg Q4W	0.4266
RAV	0.5673	BEL 10 mg/kg	0.4945	—	—	EFG 10 mg/kg	0.3011	NIP 60 mg/kg Q2W	0.4266
NIP 30/15 mg/kg Q2W	0.4763	SAT	0.4419	—	—	NIP 5 mg/kg Q4W	0.1754	ZIL 0.3 mg/kg	0.3968
SAT	0.3336	EFG 10 mg/kg	0.4037	—	—	NIP 60 mg/kg Single Dose	0.0963	INE	0.3513
RIT	0.3192	ROZ 7 mg/kg	0.2563	—	—	—	—	NIP 60 mg/kg Single Dose	0.2688
NIP 5 mg/kg Q4W	0.2937	NIP 30 mg/kg Q4W	0.2472	—	—	—	—	ROZ 7 mg/kg	0.2426
EFG 10 mg/kg	0.2858	NIP 5 mg/kg Q4W	0.1725	—	—	—	—	ROZ 10 mg/kg	0.1872
BEL 10 mg/kg	0.1891	NIP 60 mg/kg Single dose	0.0087	—	—	—	—	SAT	0.1329
NIP 60 mg/kg single dose	0.1079	—	—	—	—	—	—	—	—

Abbreviations: BAT = Batoclimab; BEL = Belimumab; ECU = Eculizumab; EFG = Efgartigimod; INE = Inebilizumab; ISC = Iscalimab; NIP = Nipocalimab; RAV = Ravulizumab; RIT = Rituximab; ROZ = Rozanolixzumab; SAT = Satralizumab; ZIL = Zilucoplan; MG‐ADL = Myasthenia Gravis Activities of Daily Living score; MGC = Myasthenia Gravis Composite score; MG‐QoL 15r = 15‐item revised Myasthenia Gravis Quality of Life score; QMG = Quantitative Myasthenia Gravis score.

#### 3.4.2 QMG

The network meta‐analysis included sixteen studies, covering 18 treatments and 29 pairwise comparisons for this outcome. Compared to placebo, BAT 340 mg (MD = −5.42; 95%CI:[−8.81, −2.02]; *p* = 0.002), BAT 680 mg (MD = −5.17; 95%CI:[−6.44, −3.89]; *p* <0.0001), ECU (MD = −2.90; 95%CI:[−4.56, −1.24]; *p* = 0.0006), INE (MD = −2.50; 95%CI:[−3.97, −1.03]; *p* = 0.0009), NIP 30/15 mg/kg Q2W (MD = −2.81; 95%CI:[−4.20, −1.42]; *p* <0.0001), RAV (MD = −2.00; 95%CI:[−3.26, −0.74]; *p* = 0.002), SAT (MD = −1.63; 95%CI:[−2.84, −0.42]; *p* = 0.008), ZIL 0.3 mg/kg (MD = −3.02; 95%CI:[−4.41, −1.64]; *p* <0.0001) significantly decreased the QMG score, whereas NIP 60 mg/kg single dose significantly increased the QMG score (MD = 2.20; 95%CI:[0.13, 4.27]; *p* = 0.04). BEL 10 mg/kg (*p* = 0.24), EFG 10 mg/kg (*p* = 0.07), NIP 30 mg/kg Q4W (*p* = 0.75), NIP 5 mg/kg Q4W (*p* = 0.88), NIP 60 mg/kg Q2W (*p* = 0.17), RIT (*p* = 0.07), ROZ 7 mg/kg (*p* = 0.48), and ZIL 0.1 mg/kg (*p* = 0.11) showed results comparable to placebo (Figure 3B and Table S2). The network diagram of this endpoint is shown in Figure 5B. BAT 680 mg was ranked best (*p*‐score = 0.96), whereas the NIP 60 mg/kg single dose regimen was ranked worst (*p*‐score = 0.009) regarding this outcome. The details of treatment ranking are provided in Table 2 and Figure S1. There was no heterogeneity among the studies included (*τ* ^2 ^< 0.0001, *I* ^2^ = 0%).

#### 3.4.3 MGC

The network meta‐analysis incorporated ten studies, involving 11 treatments and 14 pairwise comparisons for this outcome. Compared to the placebo, ECU (MD = −3.30; 95%CI:[−6.07, −0.53]; *p* = 0.01), SAT (MD = −2.99; 95%CI:[−4.65, −1.33]; *p* = 0.0004), and ZIL 0.3 mg/kg (MD = −3.37; 95%CI:[−5.33, −1.42]; *p* = 0.0007) significantly reduced the MGC score. BAT 340 mg (*p* = 0.07), BAT 680 mg (*p* = 0.06), BEL 10 mg/kg (*p* = 0.97), EFG 10 mg/kg (*p* = 0.44), RIT (*p* = 0.31), ROZ 7 mg/kg (*p* = 0.14), ZIL 0.1 mg/kg (*p* = 0.40) were comparable to placebo regarding this outcome (Figure 3C and Table S3). The network diagram of this endpoint is depicted in Figure 5C. BAT 340 mg was ranked best (*p*‐score = 0.82), whereas the BEL 10 mg/kg (*p*‐score = 0.16) was ranked worst regarding this outcome. The treatment ranking details are provided in Table 2 and Figure S2. No heterogeneity was detected among the included studies (*τ* ^2 ^< 0.0001, *I* ^2^ = 0%).

#### 3.4.4 MG‐QoL 15r

The network meta‐analysis included eleven studies, covering 14 treatments and 24 pairwise comparisons related to this outcome. Compared to placebo, BAT 340 mg (MD = −4.30;95%CI:[−8.07, −0.53]; *p* = 0.03), BAT 680 mg (MD = −4.60;95%CI:[−8.72, −0.48]; *p* = 0.03), ECU (MD = −7.10;95%CI:[−11.53, −2.67]; *p* = 0.002), NIP 30 mg/kg Q4W (MD = −4.80;95%CI:[−8.73, −0.87]; *p* = 0.02), ZIL 0.1 mg/kg (MD = −4.76;95%CI:[−8.96, −0.57]; *p* = 0.03), and ZIL 0.3 mg/kg (MD = −2.72;95%CI:[−4.68, −0.77]; *p* = 0.006) significantly reduced the MG‐QoL 15r score. EFG 10 mg/kg (*p* = 0.43), NIP 5 mg/kg Q4W (*p* = 0.86), NIP 60 mg/kg Q2W (*p* = 0.37), NIP 60 mg/kg single dose (*p* = 0.55), RAV (*p* = 0.09), RIT (*p* = 0.55), SAT (*p* = 0.13) were comparable to placebo regarding this outcome (Figure 3D and Table S4). The network diagram of this outcome is shown in Figure 5D. ECU was ranked best (*p*‐score = 0.93), whereas the NIP 60 mg/kg single dose was ranked worst (*p*‐score = 0.10) regarding this outcome. The details of the treatment ranking are given in Table 2 and Figure S3. No heterogeneity was observed across the included studies (*τ* ^2 ^< 0.0001, *I* ^2^ = 0%).

#### 3.4.5 Adverse Events

**FIGURE 7:**
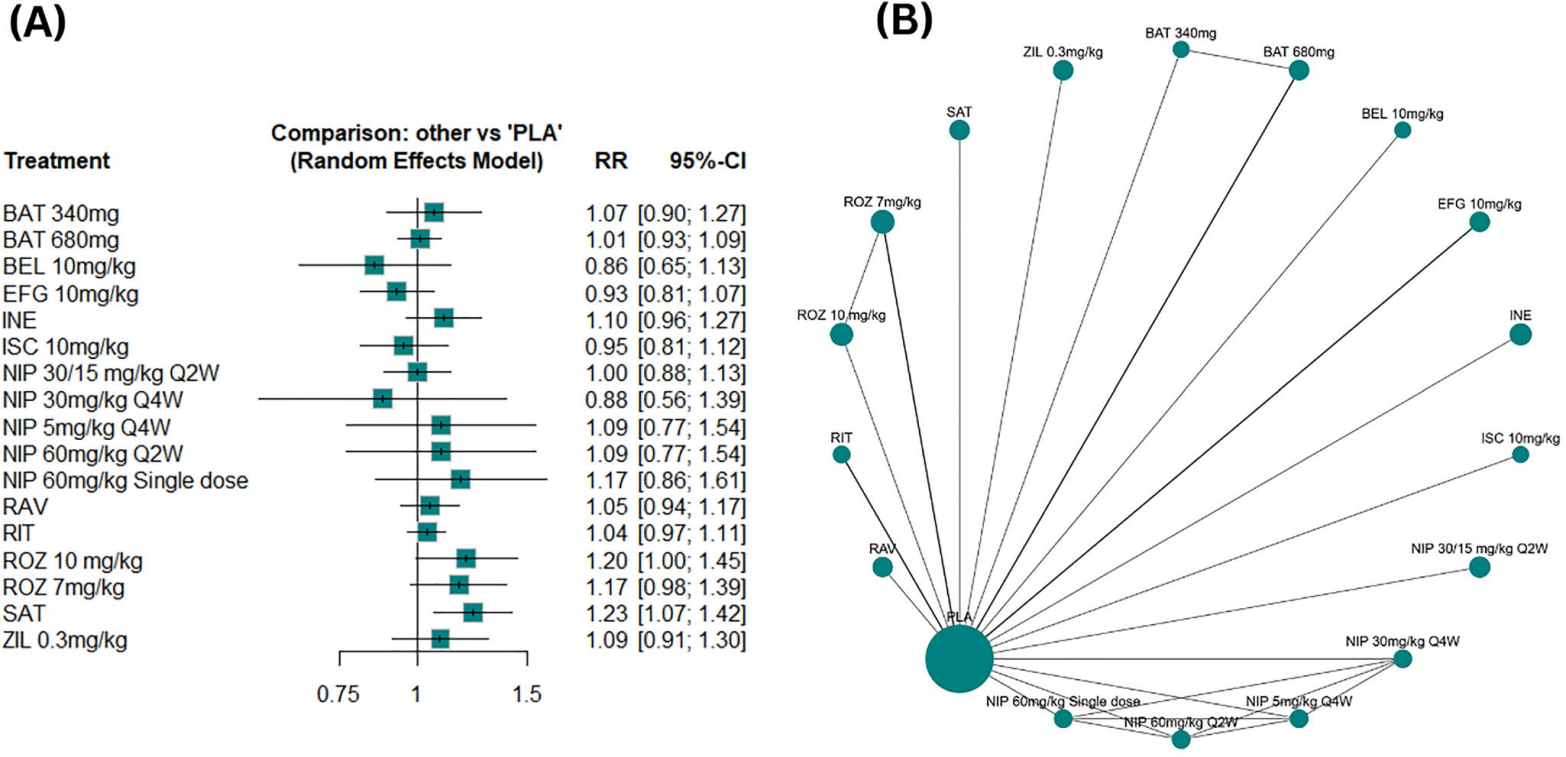
Adverse events (A) forest plot (B) network plot (BAT: Batoclimab; BEL: Belimumab; CI: Confidence interval; ECU: Eculizumab; EFG: Efgartigimod; INE: Inebilizumab; ISC: Iscalimab; MD: Mean difference; NIP: Nipocalimab; PLA: Placebo; RAV: Ravulizumab; RIT: Rituximab; ROZ: Rozanolixzumab; SAT: Satralizumab; ZIL: Zilucoplan).

The network meta‐analysis incorporated sixteen studies, evaluating 18 treatments and 29 pairwise comparisons for this outcome. Compared to placebo, only SAT significantly increased the adverse events (RR = 1.23;95%CI:[1.07, 1.42]; *p* = 0.004), whereas all other regimens were comparable to placebo (Figure 7A and Table S5). The network diagram of this outcome is shown in Figure 7B. The BEL 10 mg/kg was ranked best (*p*‐score = 0.87), whereas SAT was ranked worst (*p*‐score = 0.13). The details regarding the treatment ranking are given in Table 2 and Figure S4. No heterogeneity was found among the included studies (*τ* ^2 ^< 0.0001, *I* ^2^ = 0%).

#### 3.4.6 Publication Bias

The publication bias was assessed visually through funnel plots and statistically via Egger's regression test. No publication bias was observed upon visual inspection of the funnel plots’ symmetry, a finding that was further validated by Egger's regression test, except for the outcome of QMG score, Figures S5–S9.

## 4 Discussion

This systematic review and network meta‐analysis compared the efficacy and safety of various monoclonal antibodies in treating MG. The results showed that ROZ 10 mg/kg (Neonatal FcRn blocker) was ranked the best in reducing MG‐ADL scores, while BAT 680 mg (Neonatal FcRn blocker) was ranked best in reducing QMG scores. For MGC scores, BAT 340 mg (Neonatal FcRn blocker) was ranked best despite not reaching statistical significance, while ECU (complement inhibitor), SAT (IL‐6 receptor inhibitor), and ZIL 0.3 mg/kg (complement inhibitor) showed statistically significant decreases. In terms of quality of life, ECU (complement inhibitor) was ranked best in reducing MG‐QoL 15r scores. However, BEL 10 mg/kg (B‐lymphocyte stimulator specific inhibitor) had the lowest probability of adverse events, while SAT (IL‐6 receptor inhibitor) had the highest. Notably, the NIP 60 mg/kg single‐dose regimen (Neonatal FcRn blocker) consistently ranked poorly across multiple outcomes. Overall, these findings suggest that different monoclonal antibodies may have varying levels of efficacy and safety in treating MG, and that treatment choice may depend on the specific outcome targeted.

This meta‐analysis builds on the results of Saccà et al. (2023). Our study ranked ROZ 10 mg/kg (Neonatal FcRn blocker) as the best treatment for reducing MG‐ADL scores, while BAT 680 mg (Neonatal FcRn blocker) was ranked best for QMG scores. Sacca et al. found that anti‐FcRn treatments, as a class, showed significant improvements in MG‐ADL, QMG, and MG‐QoL scores compared to complement inhibitors. Both studies consistently suggested that monoclonal antibodies can be effective in treating MG, and different treatments may have varying levels of efficacy depending on the specific outcome being measured (Saccà et al. 2023). Another previous NMA by Gu et al. (2024) evaluated the efficacy and safety of various immunosuppressants and monoclonal antibodies in treating MG. Their results were consistent with our study and found that ROZ (Neonatal FcRn blocker) and BAT (Neonatal FcRn blocker) were effective in reducing MG‐ADL and QMG scores (Gu et al. 2024).

It should be noted that conventional immunosuppressants (e.g., steroids, azathioprine) often exhibit limited efficacy in certain MG patients and are associated with substantial adverse effects. For example, azathioprine may take 6–18 months to exert a therapeutic effect, and although initial response rates are relatively high (70%–91%), its latency limits its use in acute scenarios. Long‐term use of azathioprine, especially in conjunction with steroids, is associated with a substantial burden of adverse events: In one cohort of 163 MG patients treated over an average of 35.5 months, 61.4% experienced side effects, with 15% deemed azathioprine‐related, encompassing hematologic, gastrointestinal, infectious, and hepatic complications; severe adverse events requiring treatment cessation occurred in 6.7% (Rozsa et al. 2006). This therapeutic gap has driven the development of targeted monoclonal antibodies, which may offer better tolerability and improved clinical responses.

Current MG treatment guidelines from organizations such as the MGFA and the International MG Consensus (Narayanaswami et al. 2021) typically recommend cholinesterase inhibitors (e.g., pyridostigmine) for symptomatic relief, corticosteroids and immunosuppressants (e.g., azathioprine, mycophenolate mofetil) for long‐term disease control, IVIG or plasma exchange during acute exacerbation, and thymectomy for individuals with thymoma or early‐onset AChR+ MG. However, these traditional approaches are often suboptimal due to delayed response times (weeks to months), poor tolerability, long‐term toxicities (e.g., infections, organ toxicity, malignancy), and frequent failure to achieve sustained remission.

While FcRn inhibitors are not yet widely incorporated into standard guidelines, two agents have received regulatory approval in recent years (Li et al. 2024). Efgartigimod, the first‐in‐class FcRn antagonist, was FDA‐approved in December 2021 for adults with AChR+ generalized MG (Yang et al. 2024). ROZ secured FDA approval in June 2023 for AChR+ and MuSK+ generalized MG (Hoy 2023). Despite these advances, most global and national MG guidelines still await formal inclusion of FcRn inhibitors. Our network meta‐analysis supports both FcRn inhibitors and complement inhibitors as proven options, especially for patients who do not respond to standard immunotherapy, although more high‐quality RCTs are required to strengthen these findings. These updates should also provide guidance regarding optimal timing and patient selection, long‐term steroid tolerability, and emphasize the need for personalized treatment algorithms to determine when complement inhibition versus FcRn blockade is most appropriate.

Regarding safety and efficacy, our meta‐analysis identifies that ROZ at 10 mg/kg (Neonatal FcRn blocker) ranks highest for MG‐ADL reduction, consistent with findings from Gu et al. (2024) and Li et al. (2024), though ROZ (Neonatal FcRn blocker) is linked with increased adverse events like headaches, indicating a balance between efficacy and tolerability (Gu et al. 2024; Li et al. 2024). BAT (Neonatal FcRn blocker), at 680 mg, was best for improving QMG scores, and at 340 mg, had the highest probability for reducing the MGC score, which aligns with Gu et al. and Li et al.; however, evidence derives from a single RCT in Asian patients, limiting broader generalizability (Gu et al. 2024; Li et al. 2024). ECU emerged as the top agent for improving MG‐QoL15r scores, supported by Gu et al. (2024) and by Saccà et al. (2023), who attribute this to the ECU's complement‐inhibition mechanism. Meanwhile, BEL at 10 mg/kg (B‐lymphocyte stimulator specific inhibitor) exhibits a favorable safety profile, having the lowest reported adverse event rate, although non‐significant.

### 4.1 Limitations

This meta‐analysis has several limitations. First, the small sample sizes of existing RCTs impacted the results. Future research should include long‐term safety, maintenance efficacy, and diverse subgroups. Second, the study relied heavily on indirect comparisons, with most data drawn from comparisons between interventions and placebo, and very few, if any, direct comparisons between active treatments. Moreover, the lack of data prevented subgroup analysis by serotype (AChR/MuSK), which is known to impact response. Although we employed a random‐effects model for statistical analysis, heterogeneity in the baseline population and trial duration could act as potential confounders. There was also variation in outcome measures, which can contribute to heterogeneity. Finally, a limitation of our approach is that we evaluated different monoclonal antibodies at the product, not the class level. In clinical practice, however, only one product from each biological class is usually available. Therefore, our product‐specific rankings should be interpreted by clinicians in the context of the agents available at their institution.

## 5 Conclusion

ROZ 10 mg/kg significantly reduced the MG‐ADL score and ranked best. BAT 680 mg significantly reduced the QMG score and ranked best. BAT 340 mg had the highest probability of reducing the MGC score. ECU significantly decreased the MG‐QoL 15r and ranked best. BEL 10 mg/kg had the highest likelihood of reducing adverse events. In summary, no single monoclonal antibody demonstrates superiority across all domains (symptom relief, functional improvement, quality of life, safety), underscoring the importance of individualized therapy guided by patient‐specific priorities, including symptom burden, tolerability, and quality of life.

## Author Contributions

**Muhammad Hassan Waseem**: conceptualization, supervision, writing – review and editing. **Minahil Iqbal**: data curation, writing – original draft. **Saniya Ishtiaq**: formal analysis, writing – original draft. **Zain ul Abideen**: conceptualization, data curation, formal analysis. **Adam A. Dmytriw**: supervision, writing – review and editing. **Pawan Kumar Thada**: writing – original draft.

## Funding

The authors have nothing to report.

## Ethics Statement

The authors have nothing to report.

## Consent

The authors have nothing to report.

## Conflicts of Interest

The authors declare no conflicts of interest.

## Supporting information



Supplementary Information: brb371557‐sup‐0001‐SuppMat.docx

## Data Availability

Data can be obtained by reasonable request directed to the authors.
