# Supplementary material for: Comparing Efficacy and Safety of Various Monoclonal Antibodies in Myasthenia Gravis: A Systematic Review and Network Meta‐Analysis of Randomized Controlled Trials
Source: Brain Behav. 2026 Jun 16;16(6):e71557. doi: 10.1002/brb3.71557 (PMC13272886; doi:10.1002/brb3.71557)
Supplement: Supplementary file 1 — Supplementary Information: brb371557‐sup‐0001‐SuppMat.docx [file BRB3-16-e71557-s001.docx]

**Supplementary File**

**Index**

**Supplementary Table 1:** Detailed Search Strategies used in Different Electronic Databases

**Supplementary Table 2:** League Table for QMG

**Supplementary Table 3:** League Table for MGC

**Supplementary Table 4:** League Table for MG-QoL 15r

**Supplementary Table 5:** League Table for Adverse Events

**Supplementary Figure 1:** Rankogram for treatment ranking- QMG

**Supplementary Figure 2:** Rankogram for treatment ranking- MGC

**Supplementary Figure 3:** Rankogram for treatment ranking- MG-QoL 15r

**Supplementary Figure 4:** Rankogram for treatment ranking- Adverse Events

**Supplementary Figure 5:** Funnel Plot for MG-ADL

**Supplementary Figure 6:** Funnel Plot for QMG

**Supplementary Figure 7:** Funnel Plot for MGC

**Supplementary Figure 8:** Funnel Plot for MG-QoL 15r

**Supplementary Figure 9:** Funnel Plot for Adverse Events

| **Supplementary Table 1:** Detailed Search Strategies used in Different Electronic Databases | | |
| --- | --- | --- |
| **Database** | **Search String** | **Articles** |
| PubMed | ("Myasthenia Gravis"[MeSH] OR "Myasthenia Gravis, Ocular" OR "Ocular Myasthenia Gravis" OR "Myasthenia Gravis, Generalized" OR "Generalized Myasthenia Gravis" OR "Muscle-Specific Receptor Tyrosine Kinase Myasthenia Gravis" OR "Muscle Specific Receptor Tyrosine Kinase Myasthenia Gravis" OR "Muscle-Specific Tyrosine Kinase Antibody Positive Myasthenia Gravis" OR "Muscle Specific Tyrosine Kinase Antibody Positive Myasthenia Gravis" OR "MuSK MG" OR "MuSK Myasthenia Gravis" OR "Myasthenia Gravis, MuSK" OR "Anti-MuSK Myasthenia Gravis" OR "Anti MuSK Myasthenia Gravis" OR "Myasthenia Gravis, Anti-MuSK") AND (("Antibodies, Monoclonal"[MeSH] OR "Monoclonal Antibodies" OR "Monoclonal Antibody" OR "Antibody, Monoclonal") OR ("Fc receptor, neonatal"[MeSH] OR "Fc receptors, neonatal" OR "neonatal Fc receptors" OR "receptor, Fc neonatal" OR "FcRn neonatal transfer protein" OR "neonatal Fc receptor" OR "FCGRT protein, human" OR "FcRn protein, human" OR "Neonatal Fc receptor, human") OR ("Complement Inactivating Agents"[MeSH] OR "Agents, Complement Inactivating" OR "Inactivating Agents, Complement" OR "Complement Inhibitors" OR "Inhibitors, Complement" OR "Complement Inhibiting Agents" OR "Agents, Complement Inhibiting" OR "Inhibiting Agents, Complement" OR "Complement Inhibitor" OR "Inhibitor, Complement" OR "Complement Cytolysis Inhibiting Agents") OR ("Antigens, CD"[MeSH] OR "CD Antigens" OR "CD Antigen" OR "Antigen, CD" OR "Cluster of Differentiation Antigens" OR "Cluster of Differentiation Marker" OR "Differentiation Marker Cluster" OR "Marker Cluster, Differentiation" OR "Cluster of Differentiation Markers" OR "Cluster of Differentiation Antigen" OR "Antigen Cluster, Differentiation" OR "Differentiation Antigen Cluster" OR "Leukocyte Differentiation Antigens, Human" OR "Differentiation Antigens, Leukocyte, Human")) | **1,104** |
| **Cochrane Central** | ("Myasthenia Gravis" OR "Myasthenia Gravis, Ocular" OR "Ocular Myasthenia Gravis" OR "Myasthenia Gravis, Generalized" OR "Generalized Myasthenia Gravis" OR "Muscle-Specific Receptor Tyrosine Kinase Myasthenia Gravis" OR "Muscle Specific Receptor Tyrosine Kinase Myasthenia Gravis" OR "Muscle-Specific Tyrosine Kinase Antibody Positive Myasthenia Gravis" OR "Muscle Specific Tyrosine Kinase Antibody Positive Myasthenia Gravis" OR "MuSK MG" OR "MuSK Myasthenia Gravis" OR "Myasthenia Gravis, MuSK" OR "Anti-MuSK Myasthenia Gravis" OR "Anti MuSK Myasthenia Gravis" OR "Myasthenia Gravis, Anti-MuSK") AND (("Antibodies, Monoclonal" OR "Monoclonal Antibodies") OR ("Fc receptor, neonatal" OR "Fc receptors, neonatal" OR "neonatal Fc receptors") OR ("Complement Inactivating Agents" OR "Agents, Complement Inactivating" OR "Inactivating Agents, Complement" OR "Complement Inhibitors") OR ("Antigens, CD" OR "CD Antigens" OR "CD Antigen" OR "Antigen, CD" OR "Cluster of Differentiation Antigens")) | 177 |
| **ScienceDirect** | ("Myasthenia Gravis" OR "Generalized Myasthenia Gravis" OR "Muscle-Specific Receptor Tyrosine Kinase Myasthenia Gravis" OR "Muscle Specific Receptor Tyrosine Kinase Myasthenia Gravis" OR "MuSK Myasthenia Gravis") AND (("Antibodies, Monoclonal") OR ("Fc receptor, neonatal") OR ("Complement Inactivating Agents" ) OR ("Antigens, CD" )) | 171 |

**
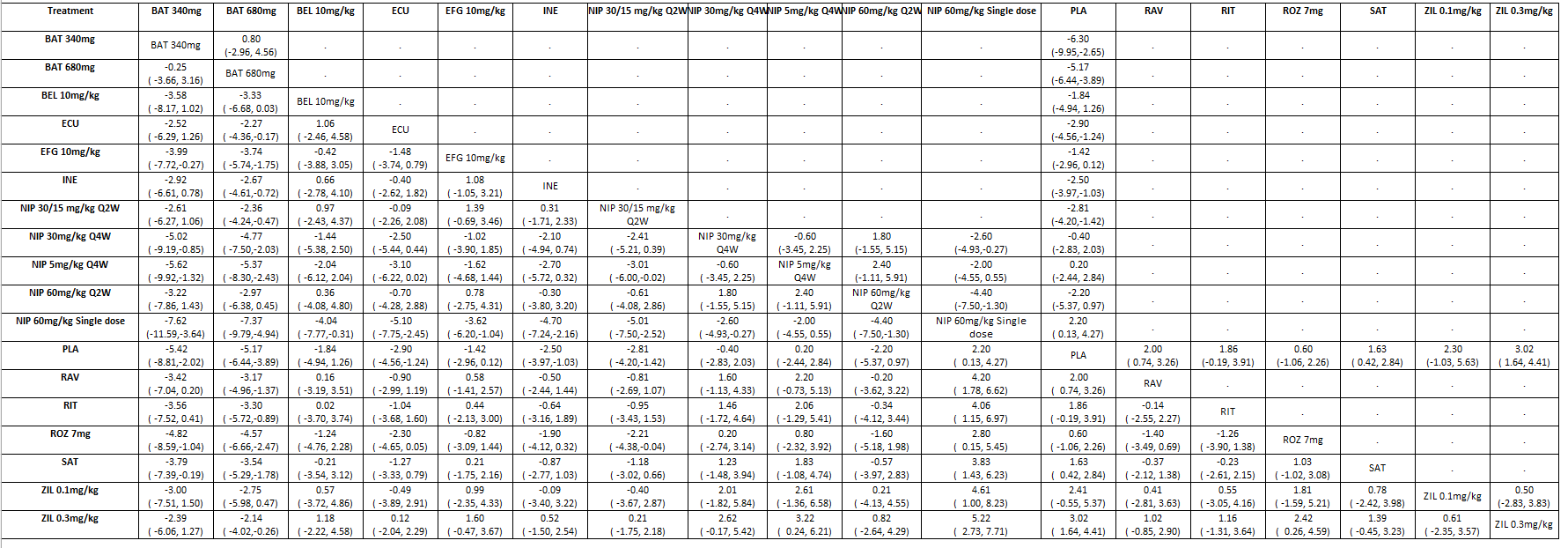
**

**Supplementary Table 2:** League Table for QMG

**
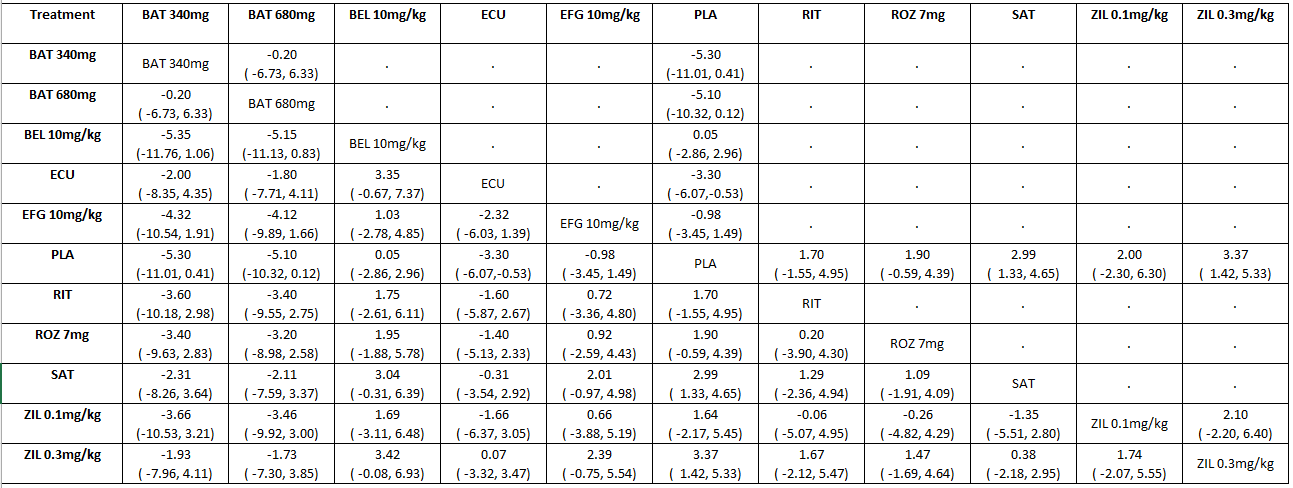
**

**Supplementary Table 3:** League Table for MGC

**
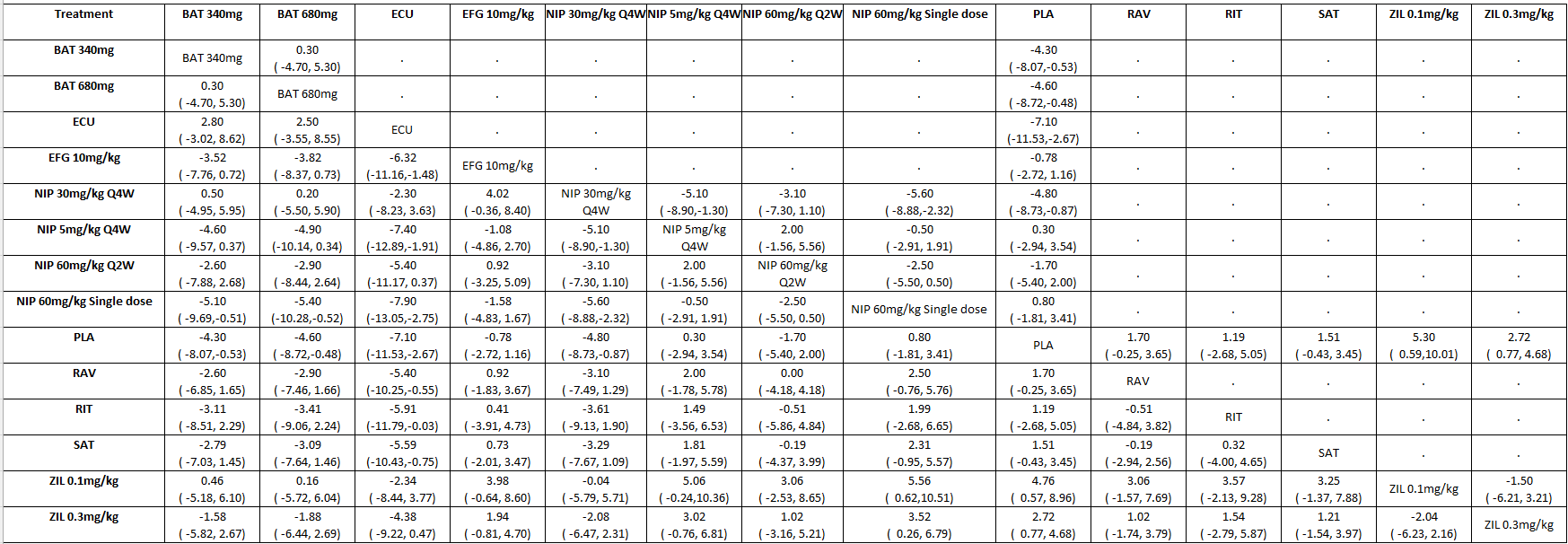
**

**Supplementary Table 4:** League Table for MG-QoL 15r

**
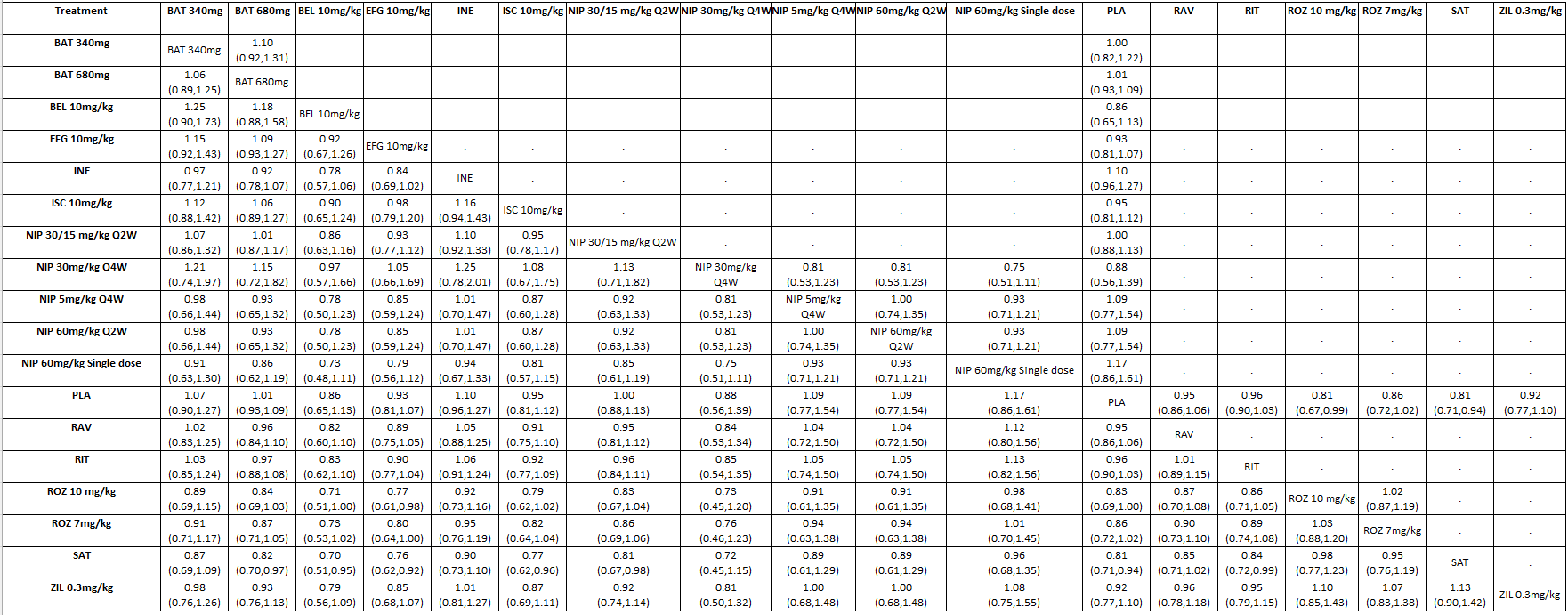
**

**Supplementary Table 5:** League Table for Adverse Events


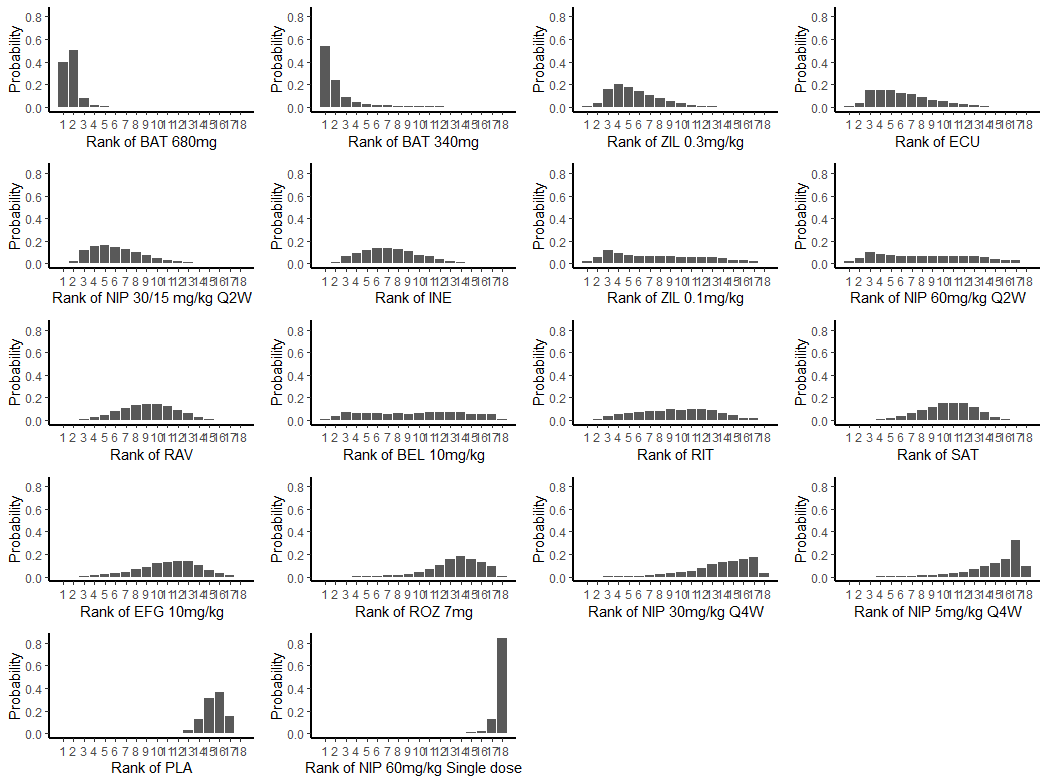


**Supplementary Figure 1:** Rankogram for treatment ranking- QMG

**
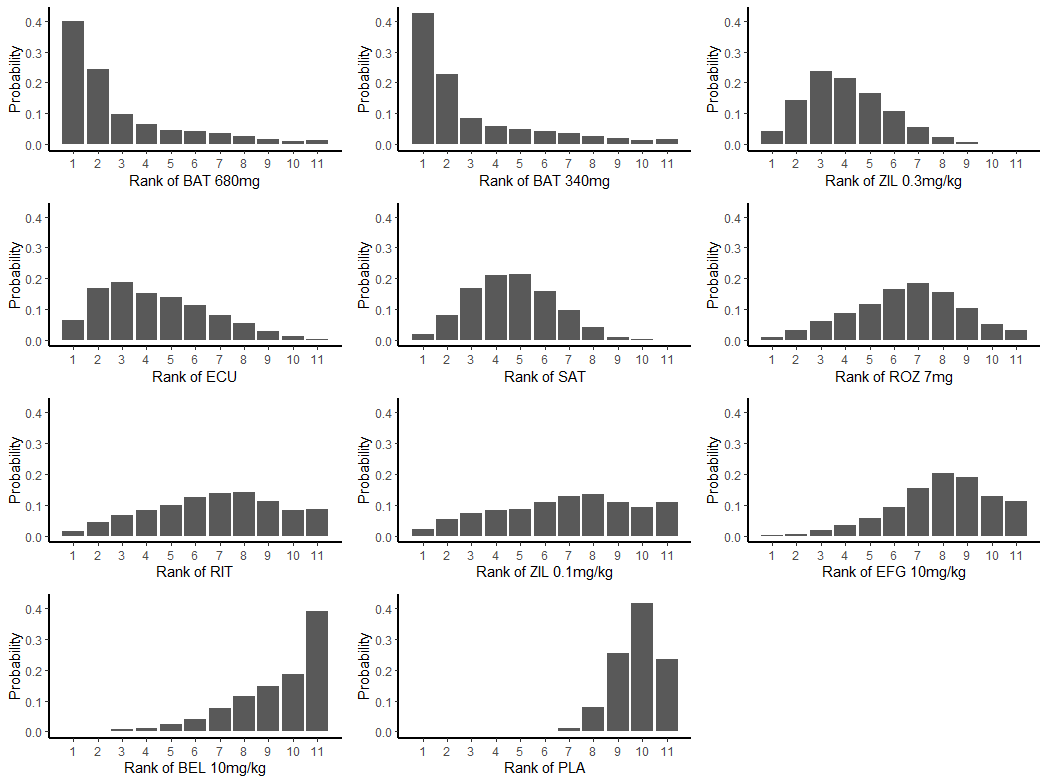
**

**Supplementary Figure 2:** Rankogram for treatment ranking- MGC

**
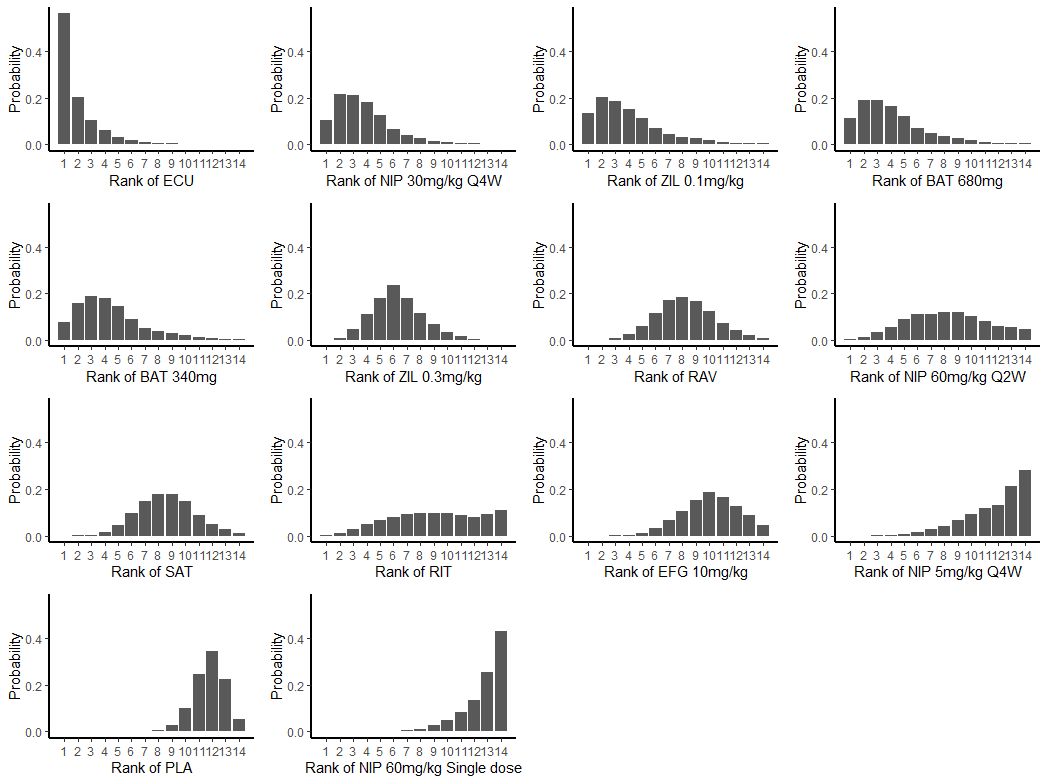
**

**Supplementary Figure 3:** Rankogram for treatment ranking- MG-QoL 15r

**
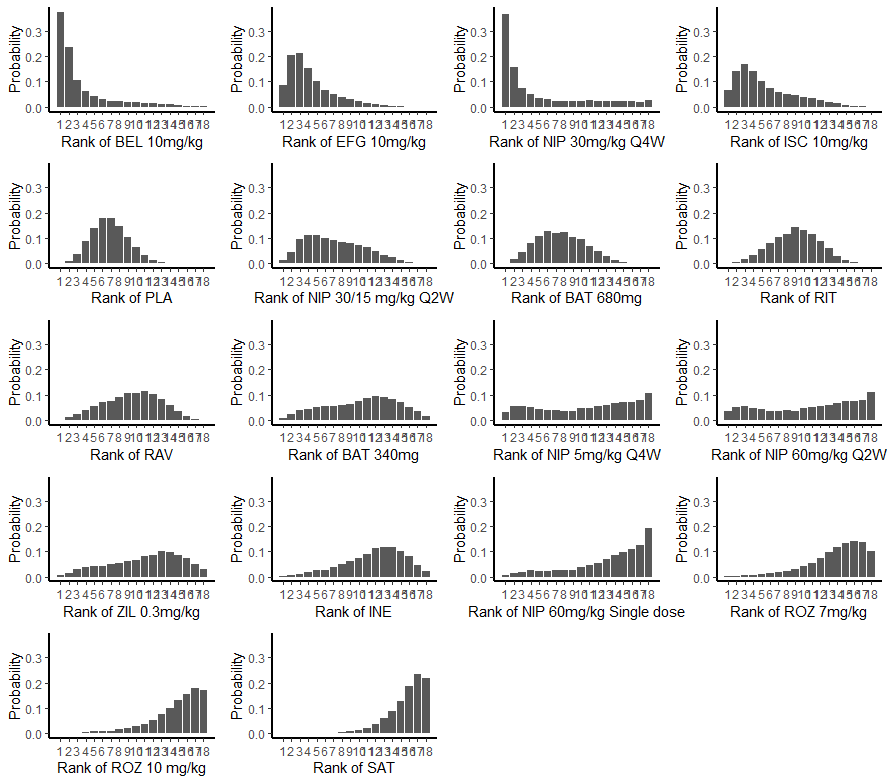
**

**Supplementary Figure 4:** Rankogram for treatment ranking- Adverse Event**
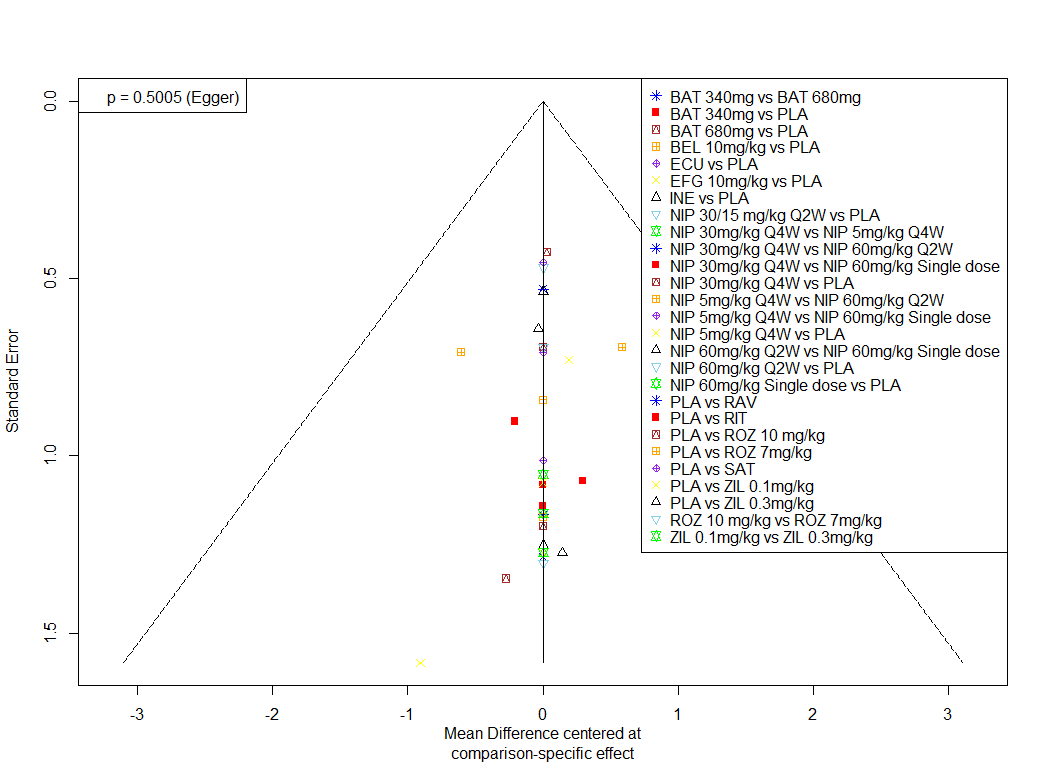
**

**Supplementary Figure 5:** Funnel Plot for MG-ADL

**
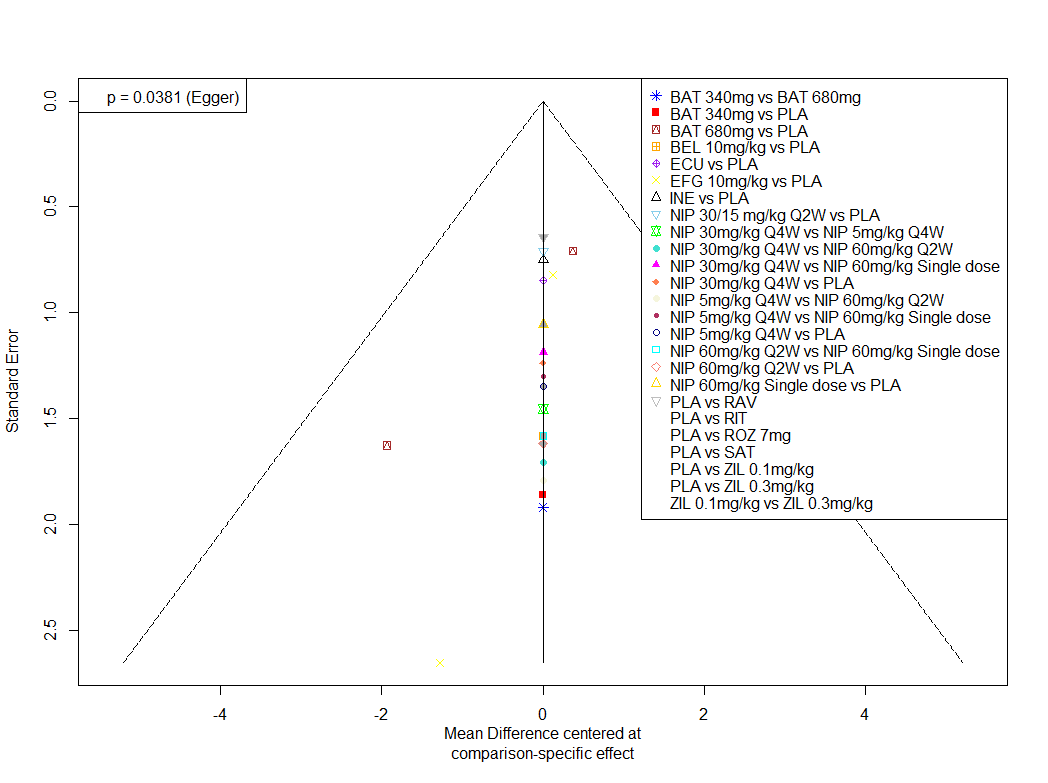
**

**Supplementary Figure 6:** Funnel Plot for QMG

**
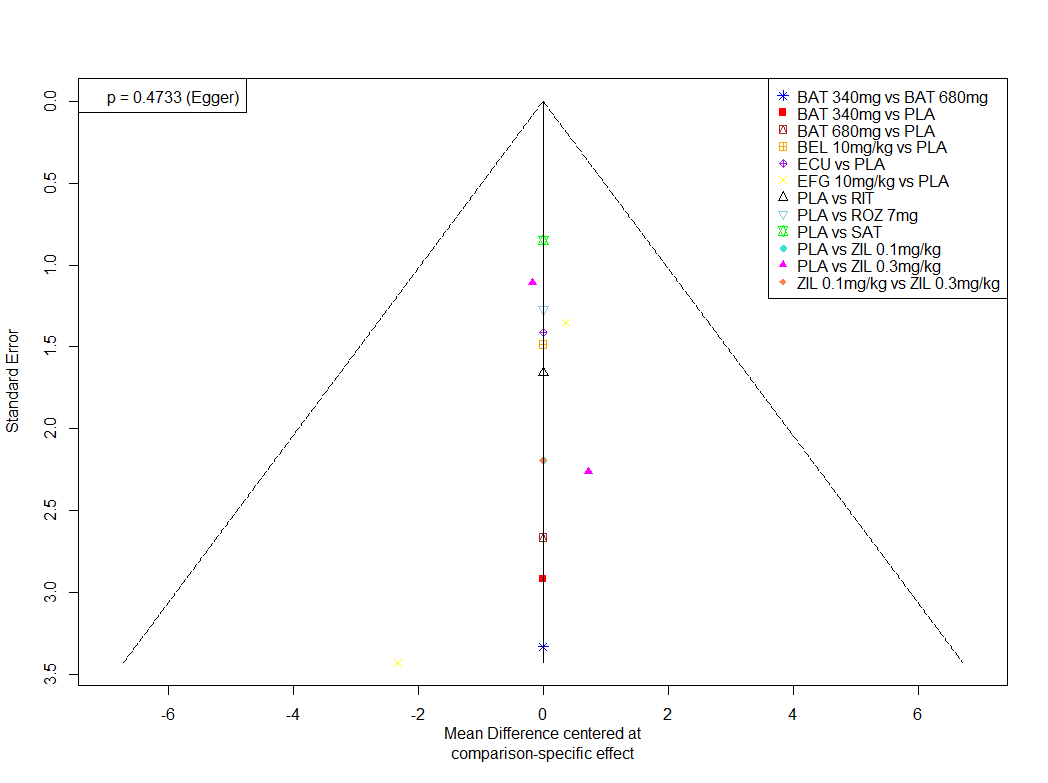
**

**Supplementary Figure 7:** Funnel Plot for MGC

**
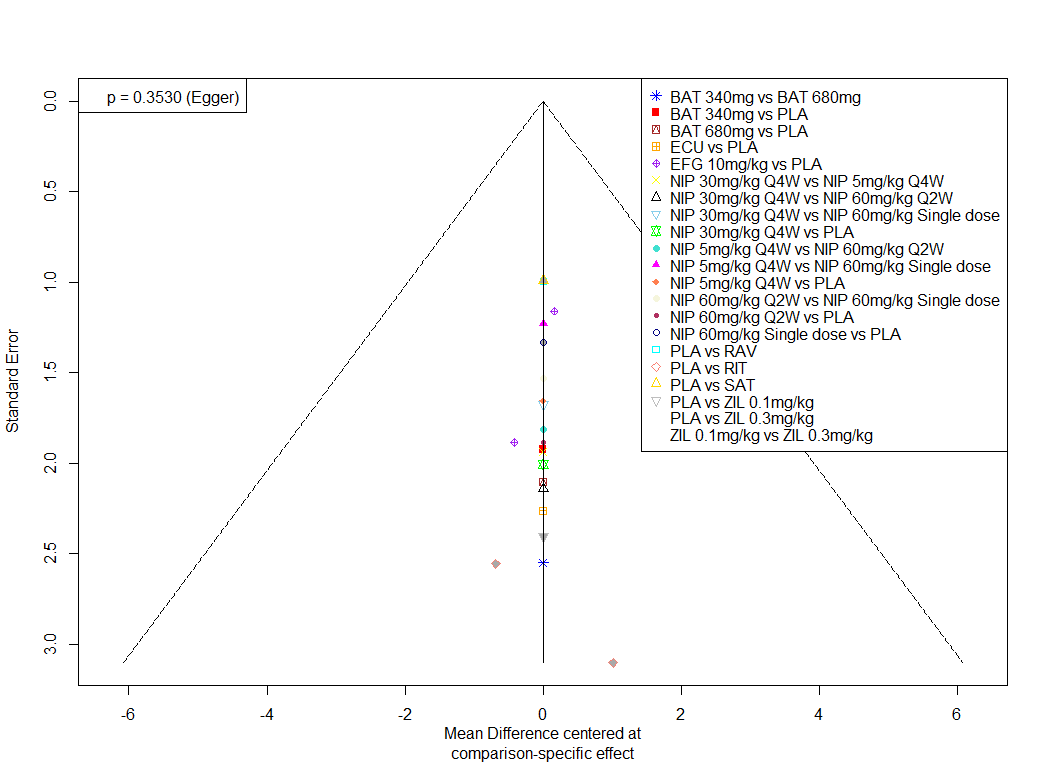
**

**Supplementary Figure 8:** Funnel Plot for MG-QoL 15r

**
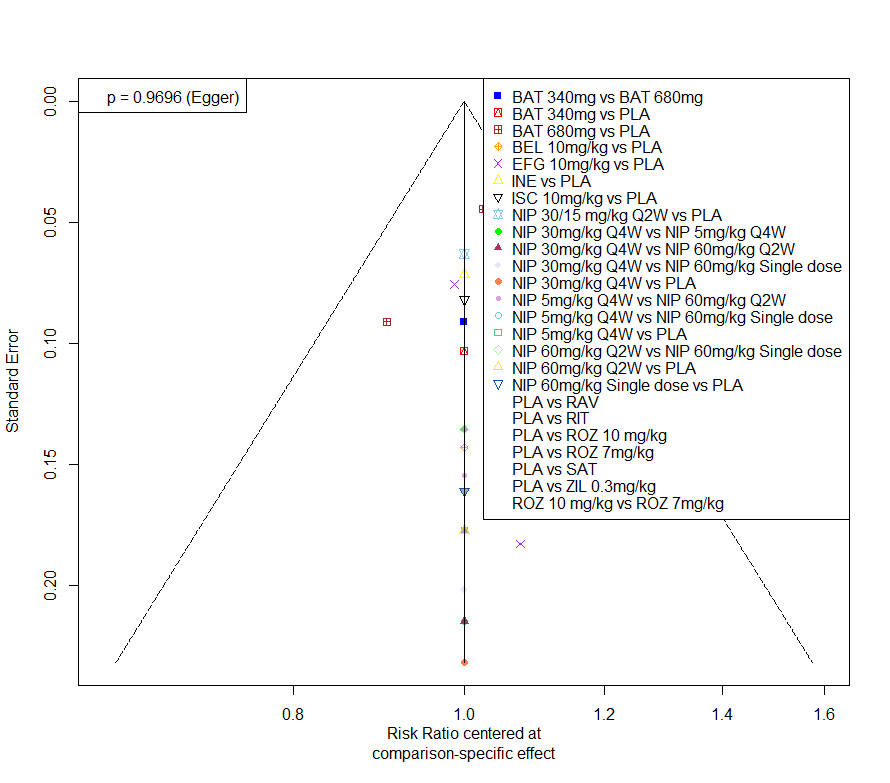
**

**Supplementary Figure 9:** Funnel Plot for Adverse Events
